# Functional deficiency of circadian regulator PER2 enhances breast cancer susceptibility and progression with immunosuppression

**DOI:** 10.1038/s41388-026-03941-3

**Published:** 2026-08-12

**Authors:** Yixin Duan, Aris T. Alexandrou, Jie Huang, Helen S. Zhang, Hongyong Zhang, Xiong Zhang, Dake Hao, Jonathan I. Berg, Ming Fan, Yili Wang, Loning Fu, Lunquan Sun, Jian Jian Li

**Affiliations:** 1 Department of Radiation Oncology, University of California Davis, Sacramento, CA USA; 2 Institute for Cancer Research, School of Basic Medical Science, Health Science Center of Xi’an Jiaotong University, Xi’an, China; 3 College of Food Science and Engineering, Ocean University of China, Qingdao, China; 4 Department of Natural and Quantitative Sciences, Holy Cross College, Notre Dame, IN USA; 5 Hangzhou Cancer Hospital, Hangzhou, China; 6 Genome Center, University of California Davis, Davis, CA USA; 7 Department of Biochemistry and Molecular Medicine, University of California Davis, Sacramento, CA USA; 8 NCI-designated Comprehensive Cancer Center, University of California Davis School of Medicine, Sacramento, CA USA; 9 Department of Surgery, University of California Davis, Sacramento, CA USA; 10 Department of Medicine/MCB, Dan L. Duncan Cancer Center, Baylor College of Medicine, Houston, TX USA; 11 Hunan Laboratory of Molecular Radiation Oncology, Xiangya Hospital, Central South University, Changsha, China

**Keywords:** Cancer epidemiology, Breast cancer, Predictive markers

## Abstract

Circadian rhythm (CR) governs systemic and tissue physiological homeostasis by regulating sleep-wake cycles, hormone secretion, and metabolism. CR disruption (CRD), such as shift work, has been associated with increased cancer risk, particularly female breast cancer (BC), yet the mechanisms underlying breast tumorigenesis and immunosuppression resulting from a specific clock gene dysfunction remain unclear. Here, we identify that circadian regulator Period 2 (PER2) is significantly downregulated in BC and correlates with poor prognosis. PER2 functional deficiency mice (*PAS-B* domain deletion, *Per2*^m/m^) display high susceptibility to DMBA/MPA-induced breast tumors. Tumor microenvironment (TME) profiling reveals that compared with *Per2*^wt^ tumors induced in *Per2*^wt^ mice, *Per2*^m/m^ tumors generated in *Per2*^m/m^ mice show reduced CD8⁺ T cells and M1-like macrophages infiltration and increased Foxp3^+^ Tregs and M2-like macrophages. Of note, reciprocal transplantation of *Per2*^m/m^ and *Per2*^wt^ tumors into *Per2*^wt^ or *Per2*^m/m^ mouse hosts reveals that systemic PER2 functional deficiency status not only boosts tumor growth but also promotes immunosuppression in TME. Consistently, *Per2* knockdown in tumor cells enhances tumor cell proliferation and immune evasion in WT hosts. Mechanistically, PER2 knockdown leads to aberrant CLOCK transactivation, which upregulates the co-expression of HER2 and CD47, thereby leading to tumor aggressive growth with limited macrophage immune checkpoint surveillance. Collectively, our findings suggest that PER2 functional deficiency-driven CLOCK-HER2/CD47 axis promotes BC susceptibility and tumor immunosuppression, which provides a potential therapeutic target to mitigate CRD-associated BC risk and aggressiveness.

**Figure:**
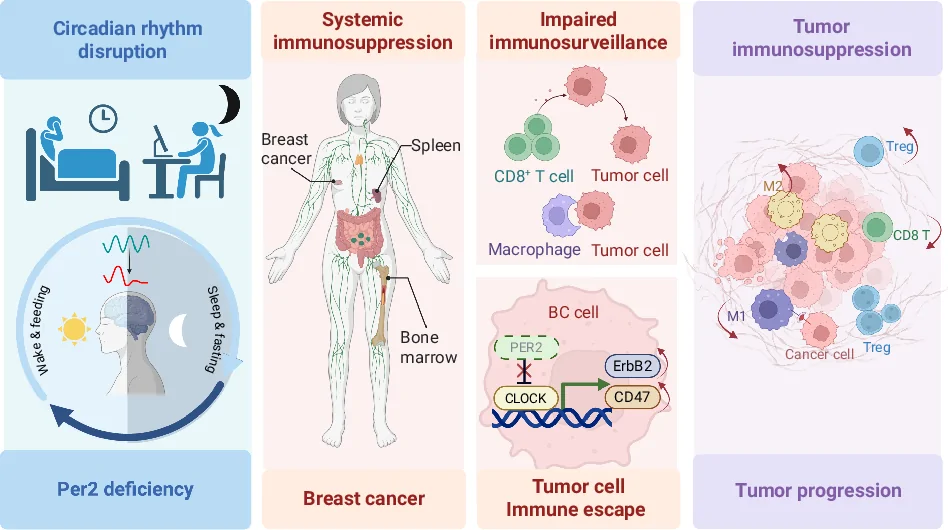

## Introduction

The circadian rhythm is an evolutionary program that controls fundamental physiological processes, including cell cycle progression, DNA repair, metabolism, and immune regulation [1–7]. Circadian rhythm disruption (CRD), whether from environmental day-night perturbations and/or dysregulation of clock genes, contributes to a broad spectrum of health issues, including the most-studied female breast malignancy [8–13]. Long-term CRD in night-shift workers has been associated with an increased risk of specific types of malignancy, including BC [14, 15]. It is also noticed that cancer patients under CRD conditions exhibit worse prognosis, enhanced drug resistance, and metastatic potential [16–18]. It is further revealed that CRD accelerates tumor aggressiveness by enhancing stem-like features, metabolic rewiring, and immunosuppressive TME [13, 18–21]. CRD-associated immunosuppressive TME has been characterized by reduced tumor-infiltrating lymphocytes (TILs) and impaired responsiveness to immune checkpoint blockade [5, 22]. Transcriptomic analyses further suggest that CRD exacerbates immune suppression by reducing T-cell abundance and enhancing immune checkpoint signaling pathways, including PD-L1 and CTLA-4 [23]. Clinical observations also indicate that the timing of ICB administration significantly influences therapeutic efficacy against melanoma [24, 25]. These findings indicate that CRD-promoted cancer susceptibility and progression are tightly associated with both intrinsic tumor growth potential and antitumor immune surveillance.

CR is maintained by interconnected transcriptional-translational feedback loops involving a cluster of core clock genes, including *CLOCK*, *BMAL1*, *PER*, *CRY*, *ROR*, and *REV-ERB* [26]. The CLOCK:BMAL1 heterodimer activates transcription of PER and CRY, which in turn represses CLOCK: BMAL1 activity to maintain the CR cycle [27]. PER2, as one of the core clock regulators, is essential for maintaining CR-guided behavioral and molecular rhythmicity in both mice and humans. Mutation of the *Per2* gene (deletion of the *PAS-B* domain) causes severe circadian rhythm disruption [28, 29]. In addition, polymorphisms in CLOCK and PER2 are associated with increased BC risk [30, 31]. PER2 downregulation is evidenced in multiple malignancies, including brain tumor [32], liver cancer [33], lymphoma [34], and leukemia [35]. PER2 is also closely involved in immune regulation, including CD4^+^ T cell differentiation [36], tumor suppression, metabolic homeostasis, and inhibition of oncogenic signaling [37]. In oral squamous cell carcinoma, lack of PER2 enhances PD-L1 expression, whereas PER2 is found to interact with HSP90 to repress PD-L1 via the NF-κB pathway [38], to sustain T cell antitumor activity. However, despite these insights, the mechanism underlying a potential integrated protumor effect of PER2 dysfunction, which simultaneously drives intrinsic tumor progression and immunosuppression, remains unelucidated. CD47, an increasingly recognized immune checkpoint protein frequently overexpressed on tumor cells, facilitates immune evasion by transmitting a “don’t eat-me” signal to macrophages, thereby suppressing tumor-eliminating phagocytosis [39]. However, it remains unclear whether PER2-associated CRD-driven tumor progression and immunosuppression is through a coordinated interplay between growth-promoting pathways and CD47 immune checkpoint dysregulation.

In this study, based on clinical data showing reduced PER2 expression in malignant tissue compared with adjacent normal tissues from BC patients, we established a carcinogen-induced BC model using the *Per2*^m/m^ mice with the *Per2*^wt^ mice as the counterpart control. We found that both PER2 functional deficiency (*Per2*^m/m^ mice) and PER2 downregulation increased BC development and induced an immunosuppressive TME. Mechanistically, PER2 functional deficiency enhanced HER2 and CD47 expression through a PER2-CLOCK-HER2/CD47 axis, which correlates with enhanced tumor aggressiveness and anti-phagocytic immune evasion.

## Methods

### Animals

WT female C57BL/6 mice (20–25 g body weight, 6–8 weeks) were purchased from Jackson Laboratory (No: 002019). C57BL/6 *Per2*-mutant (PER2 functional deficiency, *Per2*^m/m^) homozygous mice were generated and provided by the Baylor College of Medicine. This mouse model was created using homologous recombination gene targeting in embryonic stem cells, resulting in the deletion of a 2.1-kb genomic fragment that encodes the critical region of the *PAS* domain (residues 348–434, including half of the *PAS-B* subdomain and the entire *PAC* subdomain) [28, 29].

### Cell lines

C57BL/6 mouse triple-negative BC cells E0771 were provided by Professor Peter E. Fecci, Duke University. E0771 were maintained in RPMI-1640 medium supplemented with 10% FBS and 1% P/S (100 U/mL penicillin, 100 µg/mL streptomycin). Human BC cell lines, MDA-MB-231, MCF7, SKBR3, as well as THP1, and HEK-293T, were purchased from ATCC. MDA-MB-231, MCF7, SKBR3, and HEK-293T were maintained in DMEM medium supplemented with 10% FBS and 1% P/S. Human monocyte THP1 was maintained in RPMI-1640 medium supplemented with 10% FBS, 1% P/S, 10 mM sodium pyruvate, 15 mM HEPES, 4.5 g/L glucose, and 0.05 mM β-mercaptoethanol.

### Plasmids

V5-DEST-vhPER2 provided by Dr. MinGyu Lee at the University of Texas MD Anderson Cancer Center [40] was used to construct a stably PER2 overexpressing human BC cell line. pLKO.1-shPER2 purchased from Millipore Sigma (TRCN0000330732) was used to construct a stable PER2 down-regulated human BC cell line; pLKO1-puro (TCR1.5) as a control. pGL3/hCD47promoter-1554 was constructed by an established method [41]; the 1554 bp base sequence of the hCD47 promoter sequence was inserted into the vector pGL3 by HindIII/KpnI restriction endonuclease. pGL2/Her2 promoter-1030 was base constructed by an established method; the 1030 bp base sequence of the hHER2 promoter sequence was inserted into the vector pGL2 by HindIII /KpnI restriction endonuclease. PLVX-Flag-CLOCK was constructed by an established method [41]; the CLOCK cDNA (NM_001267843) was purchased from Youbio Biotechnology and inserted into the PLVX-Flag vector by Xho1/BamH1. pLenti-GFP vector (Addgene plasmid #17448) was used to generate a stably GFP-expressing BC cell line. An shRNA plasmid targeting mPer2 (TL501620) was obtained from OriGene Technologies and used to generate a stable mPer2-knockdown E0771 cell line. The pGFP-C-shLenti vector (TR30023) served as the control.

### Mouse spontaneous breast tumorigenesis

All experimental mice (WT *n* = 20, *Per2*^m/m^ *n* = 20) were gavaged weekly for six consecutive weeks with 1 mg/100 µL DMBA (Sigma-Aldrich) dissolved in olive oil. Starting from the third week of DMBA administration, 2 mg/50 µL MPA (Selleck) was subcutaneously injected every two weeks in all experimental mice. MPA was dissolved in PEG and methanol for three treatments. The administration time in all experiments was conducted between ZT0-ZT2, and mice were monitored for body weight and tumor formation twice a week and terminated when the tumor volume of experimental mice reached 1400 mm^3^; tissue samples of tumors and spleens were collected for further analysis.

### *Per2*^m/m^ subcutaneous syngeneic tumor model

Spontaneous tumors induced in *Per2*^m/m^ mice were extracted, and the tumor tissues were cut into 2–3 mm pieces before subcutaneous implantation into the syngeneic host mice (WT *n* = 10, *Per2*^m/m^ *n* = 10). After tumor implantation, tumor growth and mouse body weight were measured every 3 days. Tumor volume was calculated using the formula V = a × b² / 2(a, tumor length; b, tumor width). The experiment was terminated when the largest tumor volume in each treatment group reached 1400 mm^3^. Mice were euthanized, and tumors and spleens were collected for further analysis.

### *Per2*^wt^ (E0771) syngeneic breast tumor model

WT mice were injected with 1 × 10^6^/50 µL E0771 cells in PBS into the third mammary fat pad. When the tumor volume of experimental mice reached 1000 mm^3^, the tumor was collected and cut into 2–3 mm pieces. After anesthetizing the experimental mice (WT, *n* = 8, *Per2*^m/m^, *n* = 8), the tumor mass was implanted into the mammary fat pad and then glued to the wound. The mice’s tumor volume and body weight were measured every 3 days after the injection. The experiment was terminated when the largest tumor volume of mice in each treatment group reached 1400 mm^3^. Mice were euthanized, and the tissue of tumors and spleens was collected for further analysis.

### Hematoxylin and eosin (HE) staining

To assess intratumoral vascularization, HE-stained sections from all tumors in each experimental group were analyzed. For each tumor, five non-overlapping, representative fields of view (at 200× magnification, with a fixed field area) were systematically captured. Vascular density was quantified using ImageJ software (National Institutes of Health). Briefly, for each field, the image was calibrated, and all complete vascular lumens were manually selected based on standard morphological criteria (a clear endothelial cell lining and a visible lumen). For each sample, the three fields with the highest vascular density were selected. Within each field, the total cross-sectional area of the selected vessels was measured. The vascular density for that field was then calculated as: (Total Vascular Area/Total Field Area) × 100%. The final vascular density value for each individual tumor was reported as the mean percentage derived from its five analyzed fields.

### Immunohistochemistry (IHC)

FFPE tissue sections (4 µm) were baked, deparaffinized, and subjected to antigen retrieval in citrate buffer (pH 6.1) using microwave heating. After blocking, slides were incubated with a primary antibody overnight at 4 °C, followed by detection using the Vectastain ABC kit with a secondary antibody and a peroxidase substrate. Images were acquired on a Zeiss Axioscan Z1 and quantified using the ImmunoRatio plugin in ImageJ-Fiji. Anti-mFOXP3 (#14-5773-82, 1:100), anti-mCD8 (# PA5-81344, 1:1000), anti-mCD80 (#66406-1-Ig, 1:800), and anti-mCD206 (#18704-1-AP, 1:400) antibodies for IHC and IF staining were purchased from Invitrogen. Anti-hPER2 antibody (#DF12304) was purchased from Affinity.

Immunohistochemical scoring was performed according to previously published methods [42]. Briefly, experienced pathologists independently selected 3–6 representative regions of interest from each specimen for high-magnification imaging (200×). Protein expression was assessed using a four-tier staining intensity scale ranging from 0 to 3^+^, followed by H-score calculation based on both staining intensity and the proportion of positively stained cells. The scoring system included four categories (0/1^+^, 1^+^/2^+^, 2^+^/3^+^, and 3^+^), and the percentage of positive cells within each category was recorded. Final H-scores were calculated as the weighted sum of the percentages at each staining intensity, generating scores ranging from 0 to 300.

### RNA sequencing

TRIzol (Takara) was used to extract total RNA from DMBA/MPA-induced spontaneous tumors in WT and *Per2*^m/m^ mice. The RNA extracted from WT and *Per2*^m/m^ tumors was pooled and analyzed by RNA-seq. Enrichment analysis was performed using the phyper function in R to calculate *P* values, followed by FDR correction to obtain Q values; *Q* values ≤ 0.05 were considered significant enrichment. The volcano plot was generated from differential gene analysis, and KEGG pathway enrichment analysis was performed on up- and down-regulated differential genes. Gene set enrichment analysis (GSEA) was performed using the GSEA official package to identify a predefined gene set and assess whether there are statistically significant or consistent differences between two phenotypes. To evaluate the directional regulation of enriched terms, enrichment analysis was conducted by incorporating logFC values. For each term, a z-score was computed based on the logFC of its associated molecules, where a positive or negative z-score indicates potential positive or negative regulation, respectively. All statistical analyses and visualizations were conducted in R (version 4.2.1). The results were then visualized using the ggplot2 package, and a chord diagram was generated to present the integrated GO/KEGG enrichment analysis results combined with fold-change information.

### Flow cytometry analysis

Single-cell suspensions were prepared from the tissue by application of collagenase IV after lysis of red blood cells and rinses with 0.5% BSA/PBS. Cells were then concentrated to 1 × 10^6^ per sample in 0.5% BSA/PBS and incubated with specific antibodies for 30 min at 37 °C in the dark. Antibody titration was optimized before experimentation, with a 1:50 dilution applied. Positive and negative bead controls (1:1) were applied for channel compensation, and each sample included an FMO control. After incubation, cells were analyzed on a BD FACS Vantage, and data were processed using Flow Jo 10.0 software. The antibodies for flow cytometry, FITC-CD45 (#103122), Percp Cy5.5-CD3 (#100327), APC-CD8 (#100711), PE-CD4 (#100407), APC-Cy7-CD11b (#101225), FITC-CD80 (#104715), APC-CD206 (#141707), were purchased from Biolegend.

### Immunoblotting

Protein lysates from cells or tumor tissues were extracted using RIPA buffer supplemented with protease inhibitors (Thermo Fisher). Protein concentration was determined by BCA assay (Pierce), and 20 µg of denatured protein was separated by SDS-PAGE. Proteins were transferred to PVDF membranes using a semi-dry system, blocked with 5% non-fat milk, and probed with primary and HRP-conjugated secondary antibodies. Signal was detected by ECL (#RPN2106) and quantified using ImageJ. Anti-HER2 for WB was from Pierce (#PA5-14632), anti-CD47 for WB was from Santa Cruz (#sc-12730), anti-AKT and anti-p-AKT (ser473) for WB were from Cell signaling (#4972, #4051S), anti-mPER2 for WB was from Genetex (#GTX134478). The β-actin antibody for WB was from Sigma (#A5441).

### Real-time PCR

TRIzol was used to extract total RNA according to the manufacturer’s instructions (Takara). For cDNA synthesis, 1 µg of extracted RNA was processed with the SuperScript III cDNA Kit. RT-PCR was performed by SYBR Green PCR master mix kit according to the manufacturer’s instructions on the Real-Time PCR machine (Applied Biosystems/Thermo Fisher Scientific). The corresponding qPCR primers are listed below(5’--3’): mIFN-γ forward, CTTGGCTTTGCAGCTC TTCC; mIFN-γ reverse, GCTCATTGAATGCTTGGCGC; mTNF-α forward, GCCTATGTCTCAGCCTCTTC; mTNF-α reverse, GGAGGTTGACTTTCTCCTGG; mIL6 forward, CGGCCTTCCCT ACTTCACAA; mIL6 reverse, GGATGGTCTTGGTCCTTAGC; mTGF-β forward, TGCGCTTGCAGAGATTAAAA; mTGF-β reverse, CGTCAAAAGACAGCCACTCA; mARG1 forward, TGGC TTGCGAGACGTAGAC; mARG1 reverse, CTCCTCTGCTGTCTTCCCA; mIL10 forward, ATACTGCTAACCGACTCCT; mIL10 reverse, ATGGCCTTGTAGACACCT; mβ-actin forward, TCCTCCTGAGCGCAAGTACTCT; mβ-actin reverse, GCTCAGTAACAGTCCGCCTAGAA; hHER2 forward, GGAGAACCCCGAGTACTTGAC; hHER2 reverse, GTTCTCTGCCGTAGGTGTCC; hCD47 forward, AGAAGGTGAAACGATCATCGAGC; hCD47 reverse, CTCATCCATA CCACCGGATCT; hPER2 forward, AGGGACTGTGGCAGCACC; hPER2 reverse, TGCAGCAGGTTGAGCTGC; hβ-actin forward, CATGTACGTTGCTATCCAGGC; hβ-actin reverse, CTCCTT AATGTCACGCACGAT.

### Establishment of PER2 overexpressing and knockdown cells

For transfection of HEK-293T cells with pLenti6.3/V5-DEST-vhPER2 or pLKO.1-shPER2, using pLenti-GFP and pLKO.1-puro as control vectors, lentiviruses were generated in 293T cells according to the protocol from Addgene. The resulting PER2-overexpressing (PER2-OE) or shPER2 lentiviruses were then used to infect a human BC cell line, with a 1:1 ratio of cell culture medium (without penicillin/streptomycin) supplemented with 8 µg/mL Polybrene. Stably transfected cells were selected using a puromycin-added medium. Use RT-PCR or immunoblotting to verify gene editing status. In parallel, stable m*Per2*-knockdown E0771 cells were generated using the same lentiviral procedure with a pLenti-sh-m*Per*2 plasmid, with pLenti-GFP as the control.

### Clonogenic survival assay

For the clonogenic survival assay, cells (600 for 231, 500 for MCF7, and 800 for SKBR3) were seeded per well and incubated for 7–14 days. After culture termination, the medium was discarded, and cells were washed twice with PBS. Coomassie Brilliant Blue solution (2 mL) was then added for 30 min, followed by two rinses with PBS and additional rinsing with distilled water until clear. Colony formation was captured and counted using ImageJ software.

### Transwell invasion assay

Matrigel (#356231. BD Biosciences) was diluted with serum-free DMEM at a volume ratio of 1:1000 µL and added to the upper chamber of a 24-well Transwell and incubated for 2–4 h at 37 °C to allow gelling. 200 µL of cell suspension (2.5 × 10^4^ cells) in 1% PBS was added to the upper chamber, and 700 µL of cell culture medium with 10% FBS was added to the lower chamber. Cells were incubated for 48 h, stained using the Diff-Quick Stain kit (#K7128, IMEB INC), and analyzed under a microscope.

### Cell proliferation assay

Cells were seeded in a 96-well plate (4000 cells/ 200 µL/well) with six replicates per condition, incubating for 4 days with readings at 24, 48, 72, and 96 h. After adding 20 µL of MTS reagent to each well, cells were incubated for 3 h at 37 °C. Absorbance at 490 nm was measured with a microplate reader. Cell proliferation was determined by subtracting medium absorbance from cell absorbance.

### Immunofluorescence

FFPE sections were deparaffinized, rehydrated, and subjected to antigen retrieval. Cultured cells on coverslips were fixed with 4% PFA and permeabilized with 0.1% Triton X-100. All samples were blocked with 10% normal horse serum and incubated with double primary antibodies overnight at 4 °C. After washing, samples were incubated with fluorescent secondary antibodies (1:50) for 2 h at room temperature, treated with Sudan black, and mounted with DAPI. Imaging was performed at 200× magnification on a Zeiss Axioscan Z1, and images were analyzed using Zen 3.4. Anti-rabbit-AF488 secondary antibodies for IF were from Molecular Probes (#A11008) at a 1:200 dilution. anti-rat-AF647 (#A21247) and anti-mouse-AF488 (#A11009) secondary antibody for IF were from Invitrogen with 1:200 dilutions. Anti-rabbit Rhodamine Red secondary antibody for IF was from Immuno Research (#115035003).

### Macrophage induction and polarization

Mouse bone marrow-derived monocytes were cultured in DMEM/F12 medium with 10% FBS, 1% P/S, 5 mM L-glutamine, and 10 µM M-CSF for 5–7 days to promote cell differentiation. Cells were then seeded at 1 × 10^6^ cells per well in a 6-well plate and incubated overnight. For polarization, cells were treated with LPS (50 ng/mL) and IFN-γ (20 ng/mL) for 48 h to generate M1 macrophages, or with IL-13 (20 ng/mL) and IL-4 (20 ng/mL) for 48 h to polarize macrophages for phagocytotic analysis.

### T cell cytotoxicity

Cytotoxicity of spleen T-lymphocytes isolated from *Per2*^m/m^ and WT mice against mouse BC E0771 target cells was assessed with an LDH-cytotoxicity kit. Following co-culture for 24 h at varying effector-to-target ratios (1:10 to 1:100), LDH activity in the supernatant was quantified by measuring absorbance at 490 nm and 680 nm. The percentage of cytotoxicity was calculated as specified by the manufacturer.

### Phagocytosis assay

Macrophages, derived from bone marrow or THP-1 monocytes treated with 40 nM PMA for 48 h, were co-cultured with GFP-expressing an array of BC cells (MCF-7, SKBR3, MDA-MB-231) stably generated via lentiviral transduction using a pLenti-GFP vector, followed by puromycin selection to ensure uniform GFP expression. After 4 h of co-culture under standard conditions, cells were harvested, stained with APC-Cy7-CD11b and analyzed by flow cytometry. Phagocytic activity was measured as the proportion of CD11b^+^GFP^+^ double-positive cells, representing macrophages that had engulfed tumor-derived GFP signal.

### Luc-reporter assay

Cells were seeded at 7000 cells per well in a 96-well plate and incubated overnight. Transfection was performed using TurboFect with pGL3/hCD47 promoter-1554 or pGL2/HER2 promoter-530 luciferase reporters, and Renilla-Pol was co-transfected.III as an internal control. Cells were lysed using Luciferase Lysis Buffer, and luciferase activity was measured with a Turner TD20/20 luminometer (Promega, Madison, WI). Luciferase activity in each cell line was normalized to the Renilla signal ratio for comparative analysis.

### Chromatin immunoprecipitation quantitative PCR (ChIP-qPCR)

Chromatin immunoprecipitation (ChIP) was conducted per the manufacturer’s protocol for the Abcam kit (#ab270816). Briefly, MCF-7 cells expressing Flag-CLOCK were fixed with formaldehyde, and the chromatin was fragmented by sonication. Samples were immunoprecipitated overnight at 4 °C with anti- Flag (CST, #14793 T) or control IgG (CST, #2729S) antibody, followed by capture with protein A/G beads. After washing, bound DNA was purified and quantified by qPCR using promoter-specific primers for CD47 and HER2. Data were normalized to input and presented as fold enrichment over IgG using the 2(^–ΔΔCt^) method (CD47-promoter F: 5’-AACACAGGGTTCAGCC TCCT-3’, CD47-promoter R: 5’-CAGTCGCAGGCTCCAGAC-3’; HER2-promoter F: 5’-TGGGCAGGGCA TTTAATCTCT-3’; HER2-promoter R: 5’-AGGAGGGACTTTGGACTGGT-3’).

### Gene mutation and plasmid construction

Mutagenic oligonucleotide primers deClock185 hHER2, deClock329 hCD47 and deClock382 were designed in the QuikChange primer design platform. According to the manufacturer’s instructions, the Quik Change Lightning Site-Directed Mutagenesis Kit was applied to gene mutations, and mutant plasmids were verified by DNA sequencing. The rPER2-ΔPAS-B plasmid was generated by circular PCR mutagenesis of the V5-DEST-vhPER2 template using flanking primers (PER2-PAS-B-mut-F: 5’-AGAGTGCACTCTGGTTCAGG CGGGCAGCCTTTC-3’; PER2-PAS-B-mut-R: 5’-TGAACCAGAGTGCACTCTCTCTGCCAGCA GAAGGCAGC-3’) to delete the PAS-B domain. PCR products were DpnI-digested, self-ligated, and validated by sequencing.

### Online database analysis

Kaplan-Meier Plotter (http://kmplot.com/) was used to analyze the relationship between PER2 mRNA expression and BC prognosis. Two groups (PER2 high vs. PER2 low expression) were compared using Kaplan-Meier survival analysis, with hazard ratios and log-rank *p*-values calculated. Additionally, the TIMER database (https://cistrome.shinyapps.io/timer/) was used to compare PER2 expression between tumor and normal tissues from TCGA. Sangerbox (http://sangerbox.com/home.html) also provided data on differential PER2 expression in BC tissues, with survival analysis of high vs. low expression groups.

### Statistical analysis

All experiments were repeated three times independently, and experimental data are expressed as mean ± SE. F test to compare variances. Statistical analyses were performed using the two-tailed Student’s *t* test for comparisons between two groups or ANOVA for multiple groups. For non-parametric data, the Mann-Whitney test was used. A two-tailed Log-rank test was used to calculate the difference in the Kaplan-Meier survival curve, and Cox regression analysis was employed to develop the risk model. For survival analysis of PER2 expression in tissue microarrays, the Gehan–Breslow–Wilcoxon test was used to construct the risk model. The Gehan–Breslow–Wilcoxon test was used because it provides greater weight to early survival differences, which were prominent in our cohort. *p* values less than 0.05 were significant and indicated by asterisks as follows: *p < 0.05, **p < 0.01, ***p < 0.001, ****p < 0.0001.

### Ethics approval and consent to participate

All animal experiments were performed in accordance with relevant guidelines and regulations and were approved by the Institutional Animal Care and Use Committee (IACUC) of the University of California, Davis (approval number: IACUC 15315). Human BC tissue microarrays together with matched adjacent non-tumorous tissues (HBreD161Su01+HBreD067Su01) were obtained from Shanghai Outdo Biotech Co., Ltd. (Shanghai, China). Ethical approval for the use of these specimens was granted by the Ethics Committee of Shanghai Outdo Biotech Co., Ltd.

## Results

### PER2 functional deficiency promotes breast cancer susceptibility and aggressiveness

**Fig. 1:**
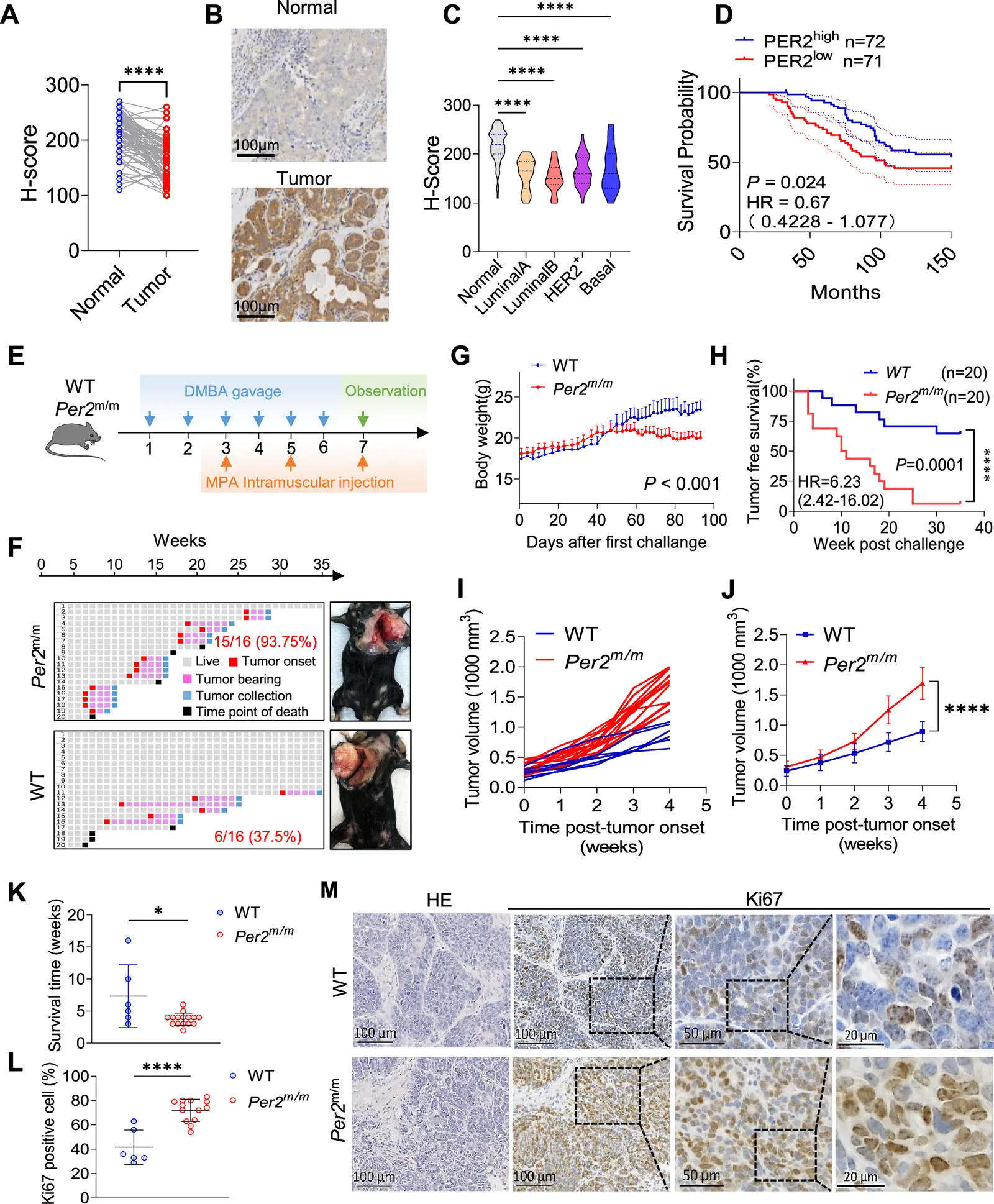
PER2 downregulation in BC and its association with tumor progression and prognosis. **A** H-score–based quantification of IHC staining demonstrates PER2 protein expression in BC tumor tissues compared with matched adjacent normal tissues (*n* = 82, Pairwise Sample t-test). **B** Representative IHC images showing PER2 protein expression in BC tumor tissues and matched adjacent normal tissues. **C** H-score–based quantification of IHC staining showing PER2 protein expression in normal breast tissues and across different BC subtypes, with statistical analysis performed using Dunn’s multiple comparisons test. **D** Kaplan–Meier analysis of overall survival (OS) in 143 BC patients stratified by high and low PER2 expression (median cutoff; Gehan–Breslow–Wilcoxon). **E** Scheme of breast tumor induction by DMBA/MPA in *Per2*^m/m^ versus WT mice. **F** Tumor incidence and disease course in individual *Per2*^m/m^ versus WT mice observed up to 35 weeks after DMBA/MPA induction (*n* = 20/group, grey: tumor-free survival, red: tumor appearance time, pink: the time before reaching the tumor volume for termination, blue: termination, black: tumor-unrelated termination with representative tumor images. Body weight (**G**), tumor-free survival (**H**, log-rank test), individual tumor volume (**I**), tumor volume (**J**, two-way ANOVA test) and tumor-carrying survival time (**K**, WT, *n* = 6; *Per2*^m/m^, *n* = 15, two-tailed Student’s *t* test) in WT and *Per2*^m/m^ mice after DMBA/MPA induction. **L, M** Quantitation and images of HE and Ki67 IHC for Ki67-expressing cells (WT tumor, *n* = 6; *Per2*^m/m^ tumor, *n* = 15, two-tailed Student’s *t* test, data shown as mean ± SD, ns, *p* > 0.05, * *p* < 0.05, ****p < 0.0001).

CRD is shown to dysregulate an array of key clock genes, including PER2 expression [43, 44], and has been associated with an increased risk of BC [31, 45–47]. The Period family genes have been implicated in multiple human cancers, and *Per2*-mutant mice exhibit increased susceptibility to radiation-induced thymocyte carcinogenesis [28, 48]. To investigate the role of PER2 in BC, we first analyzed *PER2* expression using the TCGA-BRCA dataset. *PER2* mRNA expression was significantly lower in BC tumor tissues than in matched normal tissues (Fig. S1A). Subtype analysis further revealed marked downregulation of *PER2* in the Luminal B, HER2^+^, and basal-like subtypes (Fig. S1B). Survival analysis of the TCGA-BRCA cohort demonstrated that low *PER2* expression was associated with poorer overall survival (OS; high PER2 *n* = 938 vs low PER2 *n* = 941), recurrence-free survival (RFS; high PER2 *n* = 2439 vs low PER2 *n* = 2490), disease-specific survival (DSS; high PER2 *n* = 794 vs low PER2 *n* = 313), and disease-free interval (DFI; high PER2 *n* = 459 vs low PER2 *n* = 453; Fig. S1C–F). Consistently, immunohistochemical analysis of tissue microarrays containing 82 paired BC tumor and adjacent normal tissue specimens confirmed significantly reduced PER2 protein expression in tumor tissues (Fig. 1A, B), with lower expression observed across multiple BC subtypes (Fig. 1C). Moreover, low PER2 protein expression was significantly associated with poorer OS in BC patients (high PER2 *n* = 72 vs low PER2 *n* = 71; Fig. 1D), supporting the prognostic significance of PER2 downregulation.

To determine whether PER2 functional deficiency contributes to BC development and progression, we employed the DMBA/MPA carcinogenic model [49, 50] in *Per2*^m/m^ and WT mice. *Per2*^m/m^ mice harbor a deletion of the *PAS-B* domain in *Per2*, resulting in impaired PER2-mediated repression of CLOCK:BMAL1 transcriptional activity and disrupted circadian rhythmicity [28, 29, 51]. Mice were treated weekly with oral DMBA for six weeks and alternate-week MPA injections starting at week 3 (Fig. 1E). Tumor incidence was 6/16 (37.5%) in WT mice versus 15/16 (93.75%) in *Per2*^m/m^ mice (Fig. 1F). *Per2*^m/m^ mice exhibited accelerated body weight loss, shortened tumor-free survival, and tumor-carrying survival time (Fig. 1G, H, K), and significantly larger tumor volumes (Fig. 1I, J). Histopathological analysis revealed increased tumor heterogeneity in *Per2*^m/m^ mice as evidenced by multiple tumor types (Table S1). Squamous cell carcinoma was the predominant tumor type in both groups, which suggests a different cell transformation mechanism between induced mouse BC and human BC, although this subtype is typically associated with greater malignancy and aggressiveness [52] (Fig. S1G and Table S1). Consistently, *Per2*^m/m^ tumors showed increased vascular density (Fig. S1H) and elevated Ki67 expression (Fig. 1L, M). Furthermore, in human BC cell lines (MDA-MB-231, MCF7, and SKBR3), PER2 knockdown significantly enhanced cell proliferation, whereas PER2 overexpression suppressed it (Fig. S2A–C). Consistently, Transwell assays showed that PER2 depletion increased invasive capacity, while PER2 overexpression inhibited it (Fig. S2D, E). Similarly, PER2 knockdown promoted clonogenicity, whereas PER2 overexpression reduced colony formation (Fig. S2F, G). Together, these findings support a tumor-intrinsic suppressive role for PER2 and indicate that PER2 functional deficiency promotes BC susceptibility and aggressiveness.

### Tumor immune microenvironment remodeling in PER2 functional deficiency mice

**Fig. 2:**
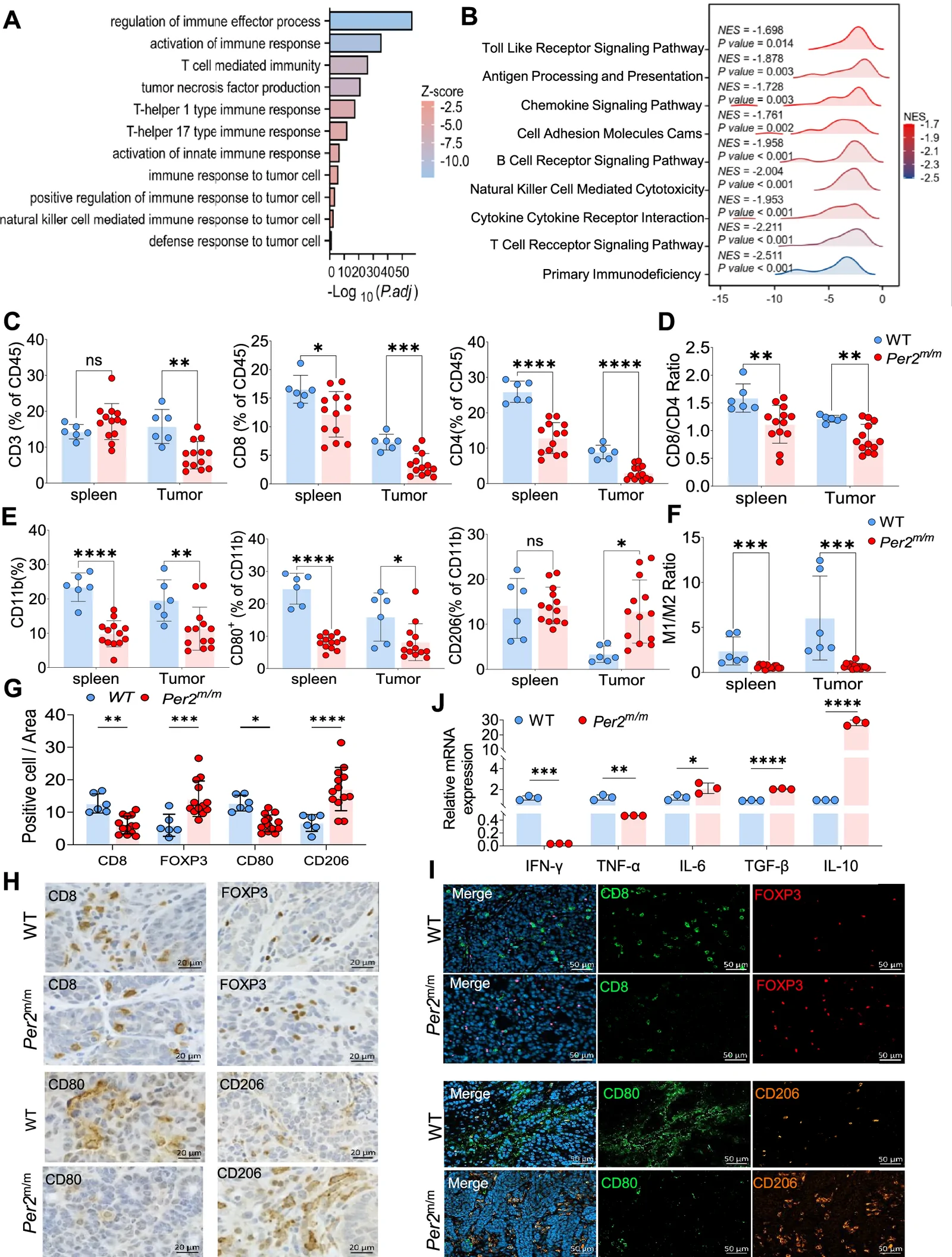
Immunosuppressive TME in *Per2*^m/m^ tumor-bearing mice. **A** Integrated GO/KEGG and FC analysis of top-downregulated pathways in tumors from *Per2*^m/m^ mice versus WT mice (negative Z-scores). **B** Gene enrichment signatures of immune-related signaling pathways in tumors from *Per2*^m/m^ mice versus WT mice. **C** Flow cytometry analysis of CD3^+^ T cells (left), CD8^+^ T cells (middle), and CD4^+^ T cells (right) in the spleen and tumors of WT and *Per2*^m/m^ DMBA-carcinogenic mice. **D** CD8^+^/CD4^+^ T cells ratio in the spleen and tumors of WT and *Per2*^m/m^ DMBA-carcinogenic mice. **E** Flow cytometry of CD11b^+^ monocytes (left), CD80^+^ M1 (middle), and CD206^+^ M2 macrophages (right) in the spleens and tumors of WT and *Per2*^m/m^ DMBA-carcinogenic mice. **F** CD80^+^ M1/CD206^+^ M2 ratio in the spleen and tumors of WT and *Per2*^m/m^ DMBA-carcinogenic mice. Representative IHC quantitation (**G**) and images (**H**) of CD8^+^ T cells, FOXP3^+^ Treg cells, CD80^+^ M1 macrophages, and CD206^+^ M2 macrophages in DMBA-induced tumors of WT and *Per2*^m/m^ mice. **I** mRNA expression of cytokines IFN-γ, TNF-α, TGF-β, IL-6, and IL-10 in tumors induced in WT and *Per2*^m/m^ mice. **J** Representative immunofluorescence images of infiltrated CD8^+^ and FOXP3^+^ or CD80^+^ and CD206^+^ cells in the tumor induced in WT and *Per2*^m/m^ mice tumor. In **C–J**, WT mice *n* = 6, *Per2*^m/m^ mice *n* = 15, data shown as mean ± SD, two-tailed Student’s *t* test, ns, *p* > 0.05, *p < 0.05, **p < 0.01, ***p < 0.001, ****p < 0.0001.

To further determine whether PER2 functional deficiency-mediated BC is associated with alterations in the immune microenvironment, we compared the transcriptomic profiles of *Per2*^m/m^ and *Per2*^wt^ tumors and identified a set of immune-related genes that were downregulated in *Per2*^m/m^ tumors. GO and KEGG enrichment analyses showed that the downregulated genes in *Per2*^m/m^ tumor were enriched in anti-tumor immune pathways, particularly immune effector processes and T cell–mediated immunity. (Fig. 2A). Consistently, gene set enrichment analysis (GSEA) further confirmed the suppression of immune recognition, activation, and cytotoxic effector functions in *Per2*^m/m^ tumors (Fig. 2B). Collectively, these analyses indicate that PER2 functional deficiency is associated with the establishment of a profoundly immunosuppressive TME that may contribute to enhanced tumor aggressiveness.

Consistent with the immunosuppressive transcriptomics results, flow cytometry analyses of TILs revealed reduced abundance of CD3^+^, CD4^+^, and CD8^+^ T cells in *Per2*^m/m^ tumors. Importantly, this reduction was accompanied by lower frequencies of splenic CD4^+^ and CD8^+^ T cells in tumor-bearing mice (Fig. 2C). In addition, the CD8/CD4 ratio, a prognostic immune index in BC [53], was also reduced in both tumors and spleens (Fig. 2D). Moreover, CD11b^+^ myeloid cells and CD80^+^ M1-like macrophages were decreased, whereas CD206^+^ M2-like macrophages were increased in *Per2*^m/m^ tumors, resulting in a reduced M1/M2 ratio (Fig. 2E, F). Consistent with these findings, immunohistochemistry and immunofluorescence staining confirmed reduced intratumoral CD8^+^ T cells and CD80^+^ M1-like macrophages, alongside expansion of FOXP3^+^ Tregs and CD206^+^ M2-like macrophages (Fig. 2G–I). Cytokine profiling further supported this immunosuppressive milieu, with *Per2*^m/m^ tumors exhibiting decreased expression of IFN-γ and TNF-α, and increased TGF-β and IL-10 levels (Fig. 2J). Together, these findings indicate that impaired *Per2* function promotes an immunosuppressive TME.

### Host and tumor-intrinsic PER2 alterations cooperatively contribute to tumor immunosuppression

**Fig. 3:**
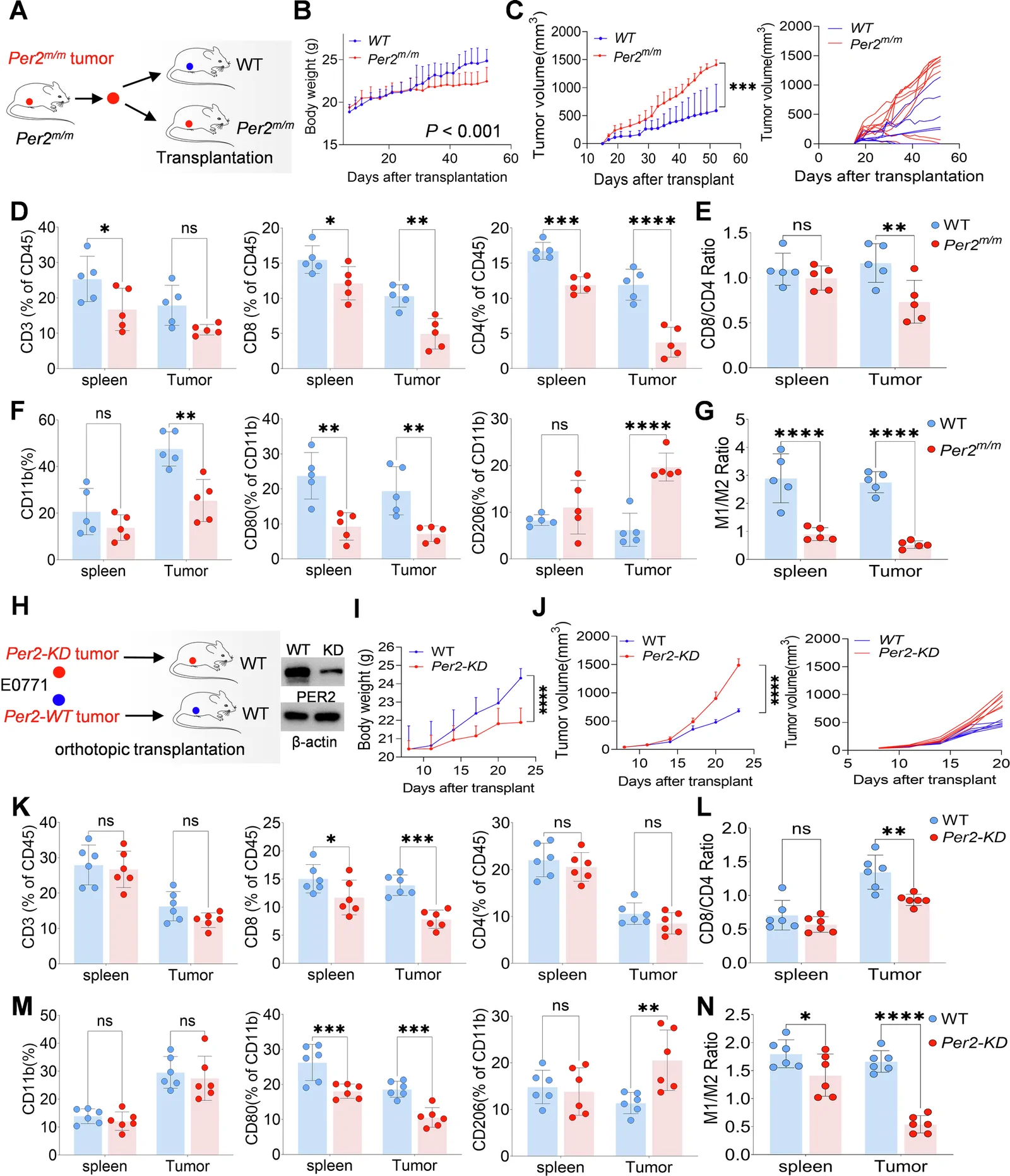
PER2 functional deficiency drives immune suppression and tumor progression. **A** Scheme of DMBA/MPA-induced *Per2*^m/m^ tumor transplantation into WT or *Per2*^m/m^ recipient mice. Body weight (**B**) and tumor growth (**C**) of WT and *Per2*^m/m^ mice bearing transplanted *Per2*^m/m^ tumors (WT mice *n* = 5, *Per2*^m/m^ mice *n* = 7, two-way ANOVA test). **D** Flow cytometry analysis of tumor CD3^+^ T cells (left), CD8^+^ T cells (middle), and CD4^+^ T cells (right) in spleens and tumors of WT and *Per2*^m/m^ mice with transplanted *Per2*^m/m^ tumors. **E** CD8^+^/CD4^+^ T cells ratio in the spleens and tumors of WT and *Per2*^m/m^ mice bearing *Per2*^m/m^ tumors. Flow cytometry analysis of the tumor CD11b^+^ monocytes (**F**, left), CD80^+^ M1 macrophages (**F**, middle), and CD206^+^ M2 macrophages (**F**, right) and CD80^+^ M1/CD206^+^ M2 ratio (**G**) in the spleens and tumors of WT and *Per2*^***m/m***^ mice bearing transplanted *Per2*^m/m^ tumors (WT mice *n* = 5, *Per2*^m/m^ mice *n* = 5, two-tailed Student’s *t* test). **H** Schematic illustration of syngeneic E0771 mouse tumor models established by transplantation of E0771 *Per2*-KD or *Per2*-WT cells into WT mice, with corresponding PER2 expression levels indicated in *Per2*-WT and *Per2*-KD cells. Body weight (**I**) and tumor growth (**J**) was measured (*n* = 6, two-way ANOVA test). Flow cytometry analysis of tumor CD3^+^ (left), CD8^+^ (middle), and CD4^+^ T cells (right) in the spleens and tumors (**K**) and the CD8^+^/CD4^+^ cell ratio (**L**) in the spleens and tumors of WT mice bearing E0771 *Per2*-KD and *Per2*-WT tumors. **M** Flow cytometry analysis of tumor CD11b^+^ monocytes (left), CD80^+^ M1 (middle), and CD206^+^ M2 macrophages (right) in the spleens and tumors of WT mice bearing E0771 *Per2*-KD and *Per2*-WT tumors. **N** CD80^+^ M1/CD206^+^ M2 macrophages ratio in spleen and tumors of WT mice bearing *Per2*-KD and *Per2*-WT tumors (WT mice *n* = 6, two-tailed Student’s *t* test). In **C–G**, **I–N**, data shown as mean ± SD, ns, *p* > 0.05, *p < 0.05, **p < 0.01, ***p < 0.001, ****p < 0.0001.

We next tested whether PER2 functional deficiency drives immune remodeling that shapes an immunosuppressive TME and promotes BC progression. Syngeneic *Per2*^m/m^ tumors derived from tumor-bearing *Per2*^m/m^ donor mice were transplanted into WT or *Per2*^m/m^ recipient hosts (Fig. 3A). Notably, *Per2*^m/m^ mice exhibited accelerated body weight loss and enhanced tumor growth (Fig. 3B, C). Flow cytometry analyses further revealed reduced infiltration of CD3^+^, CD4^+^, and CD8^+^ T cells and CD80^+^ M1 macrophages, together with increased CD206⁺ M2-like macrophages, resulting in lower CD8/CD4 and M1/M2 ratios in *Per2*^m/m^ tumors regrowth in *Per2*^m/m^ hosts (Fig. 3D–G).

We then repeated the experiment using *Per2*^wt^ E0771 tumors and transplanted them into *Per2*^m/m^ and WT mice. Again, *Per2*^m/m^ hosts carrying *Per2*^wt^ E0771 tumors exhibited increased body weight loss and faster tumor growth compared to the WT hosts carrying the same tumors (Fig. S3A–C). Similarly, tumors growing in *Per2*^m/m^ hosts showed reduced infiltration of CD3^+^, CD4^+^, and CD8^+^ T cells and CD80^+^ M1 macrophages, increased CD206^+^ M2 macrophages, and lower intratumoral CD8/CD4 and M1/M2 ratios (Fig. S3D–G). Tumor tissue histological analyses confirmed reduced CD8⁺ T cells and M1 macrophages, accompanied by increased FOXP3⁺ Tregs and CD206^+^ M2 macrophages in *Per2*^m/m^ host tumors (Fig. S3H–J). Consistently, *Per2*^wt^ tumors growing in *Per2*^m/m^ hosts exhibited lower expression of IFN-γ and TNF-α, alongside elevated TGF-β and IL-10 levels (Fig. S3K). These results indicate that, following transplantation of identical *Per2*^m/m^ or *Per2*^wt^ tumors, systemic PER2 functional deficiency promotes an immunosuppressive host environment that facilitates tumor progression, indicating a potential tumor-host communication.

While transplantation of *Per2*^m/m^ or *Per2*^wt^ tumors into *Per2*^m/m^ and WT hosts revealed a potential critical host-dependent difference in tumor immunity, the possible contribution of tumor-intrinsic *Per2* downregulation remained unclear. To address this issue, we generated *Per2* knockdown (*Per2*-KD) E0771 cells and established an orthotopic BC model in WT mice (Fig. 3H). Mice bearing *Per2*-KD tumors exhibited accelerated tumor growth and more rapid body weight loss compared to WT tumors (Fig. 3I, J). Flow cytometry analyses further showed that, although the proportions of CD3^+^, CD4^+^, and CD11b^+^ cells were not significantly altered in either tumors or spleens, *Per2*-KD tumors displayed reduced infiltration of CD8^+^ T cells and CD80^+^ M1 macrophages, accompanied by increased CD206^+^ M2 macrophage infiltration, resulting in decreased CD8/CD4 and M1/M2 ratios (Fig. 3K–N). Collectively, these results demonstrate that systemic PER2 functional deficiency promotes an immunosuppressive TME and accelerates tumor progression, whereas tumor-intrinsic PER2 downregulation further contributes to this phenotype, highlighting a cooperative role of host immunity and tumor-intrinsic PER2 alterations in BC progression.

### PER2 functional deficiency impairs T cell and macrophage function in tumor-free immune organs

**Fig. 4:**
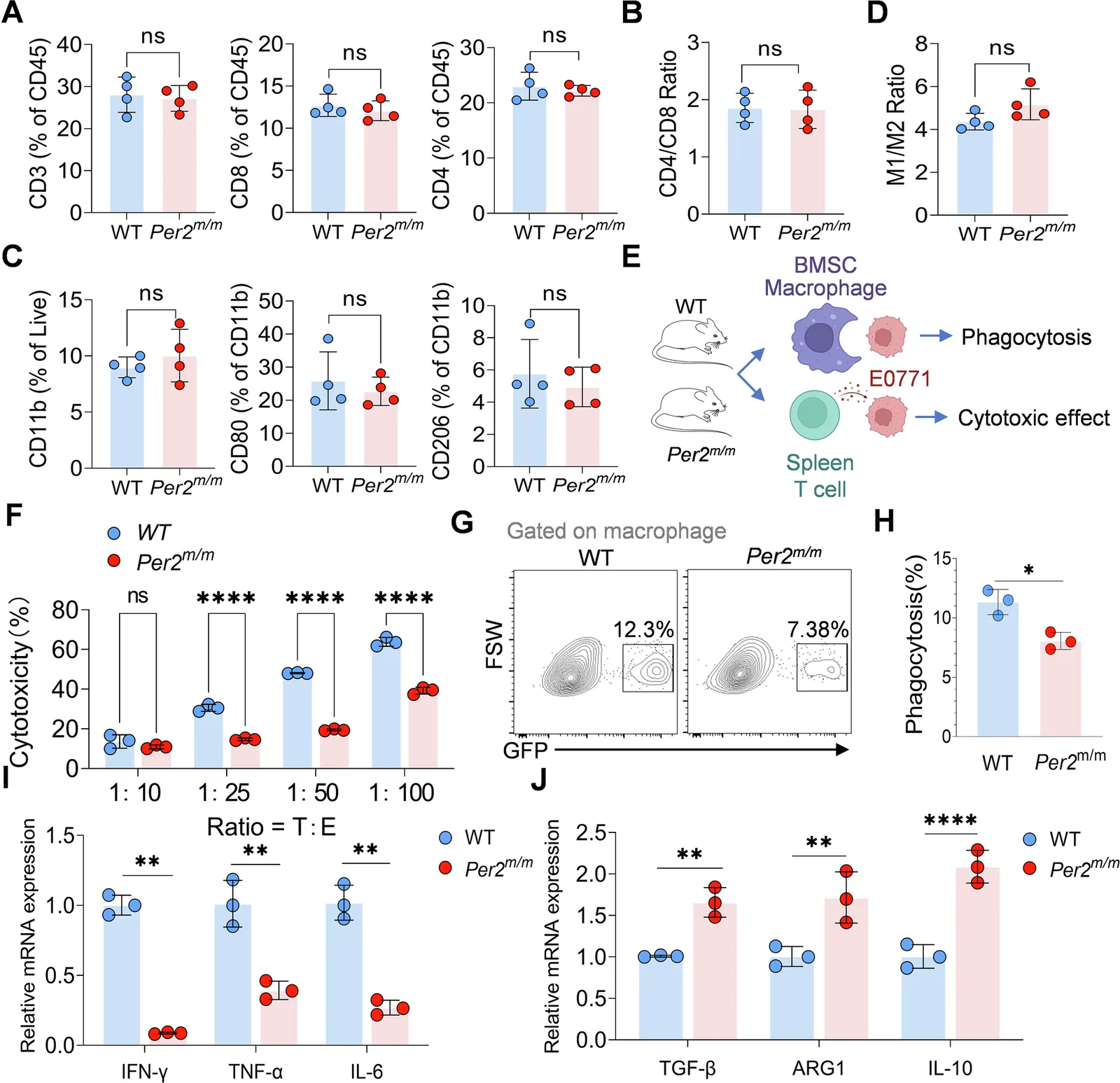
PER2 functional deficiency diminishes T cell and macrophage activity in tumor-free immune organs. Flow cytometry analysis of CD3^+^, CD8^+^, CD4^+^ T cells (**A**), CD8^+^/CD4^+^ T cell ratio (**B**), CD11b^+^, CD206^+^, CD80_+_ macrophages (**C**), and CD80^+^ M1/CD206^+^ M2 macrophage ratio (**D**) in the spleen of non-tumor-bearing WT and *Per2*^m/m^ mice (WT mice *n* = 4, *Per2*^m/m^ mice *n* = 4, two-tailed Student’s *t* test). **E** Schematic of the in vitro co-culture system to assess the phagocytic and anti-tumor functions of macrophages and T cells from WT and *Per2*^m/m^ mice. **F** Cytotoxicity on targeted mouse BC E0771 cells was measured with T cells isolated from spleens of WT and *Per2*^m/m^ mice with increasing ratio of target tumor cells (T) to effector T cells (E), *n* = 3, two-way ANOVA test. **G** Phagocytosis on E0771 cells by macrophages derived from WT and *Per2*^m/m^ mice. **H** Quantitation of macrophage phagocytic ability in (**G**) (*n* = 3, two-tailed Student’s *t* test). mRNA levels of IFN-γ, TNF-α and IL-6 in M1 (**I**) and TGF-β, ARG1 and IL-10 in M2 (**J**) macrophages from WT and *Per2*^m/m^ mice (*n* = 3, two-tailed Student’s *t* test). In **A–J**, data shown as mean ± SD, ns, *p* > 0.05; *p < 0.05; **p < 0.01; ***p < 0.001; ****p < 0.0001.

Given the reduced TILs and weakened antitumor immune response observed in tumor-bearing *Per2*^m/m^ mice, we further investigated whether systemic PER2 functional deficiency affects immune cell distribution or function under tumor-free conditions. To address this, we analyzed splenic T cell and macrophage populations in tumor-free WT and *Per2*^m/m^ mice, as the spleen is a major peripheral immune organ involved in systemic immune regulation [54]. Surprisingly, no significant differences were observed in T cell or myeloid cell frequencies, nor in CD8/CD4 or M1/M2 ratios, between WT and *Per2*^m/m^ mice when both samples were simultaneously collected at the same circadian time point, 8:00 a.m. (Fig. 4A–D). These findings suggest that PER2 functional deficiency alone is insufficient to alter splenic immune cell distribution, despite the increased tumor susceptibility observed in *Per2*^m/m^ mice.

Based on these findings, we speculated that the enhanced BC susceptibility and tumor aggressiveness of *Per2*^m/m^ mice may be due to impaired antitumor immune function. To test this hypothesis, we assessed the antitumor functions of T cells and macrophages derived from tumor-free WT and *Per2*^m/m^ mice by co-culturing splenic T cells or bone marrow-derived macrophages with syngeneic E0771 mouse BC cells (Fig. 4E). Indeed, T cells from *Per2*^m/m^ mice exhibited significantly reduced cytotoxicity against syngeneic E0771 BC cells, as measured by LDH release (Fig. 4F). Similarly, macrophages derived from *Per2*^m/m^ mice displayed diminished phagocytic capacity together with a bias toward M2-like polarization, whereas WT macrophages displayed a more M1-like phenotype (Fig. 4G–J). These findings are consistent with the increased M2-like macrophage infiltration observed in *Per2*^m/m^ tumors (Fig. 2E). Collectively, these results indicate that although the frequencies of peripheral T cells and macrophages remain largely unchanged under tumor-free conditions, immune cells from *Per2*^m/m^ mice exhibit impaired antitumor immune function, thereby potentially contributing to enhanced BC susceptibility and progression.

### PER2 functional deficiency activates ErbB (HER)–AKT signaling and promotes immunosuppression

**Fig. 5:**
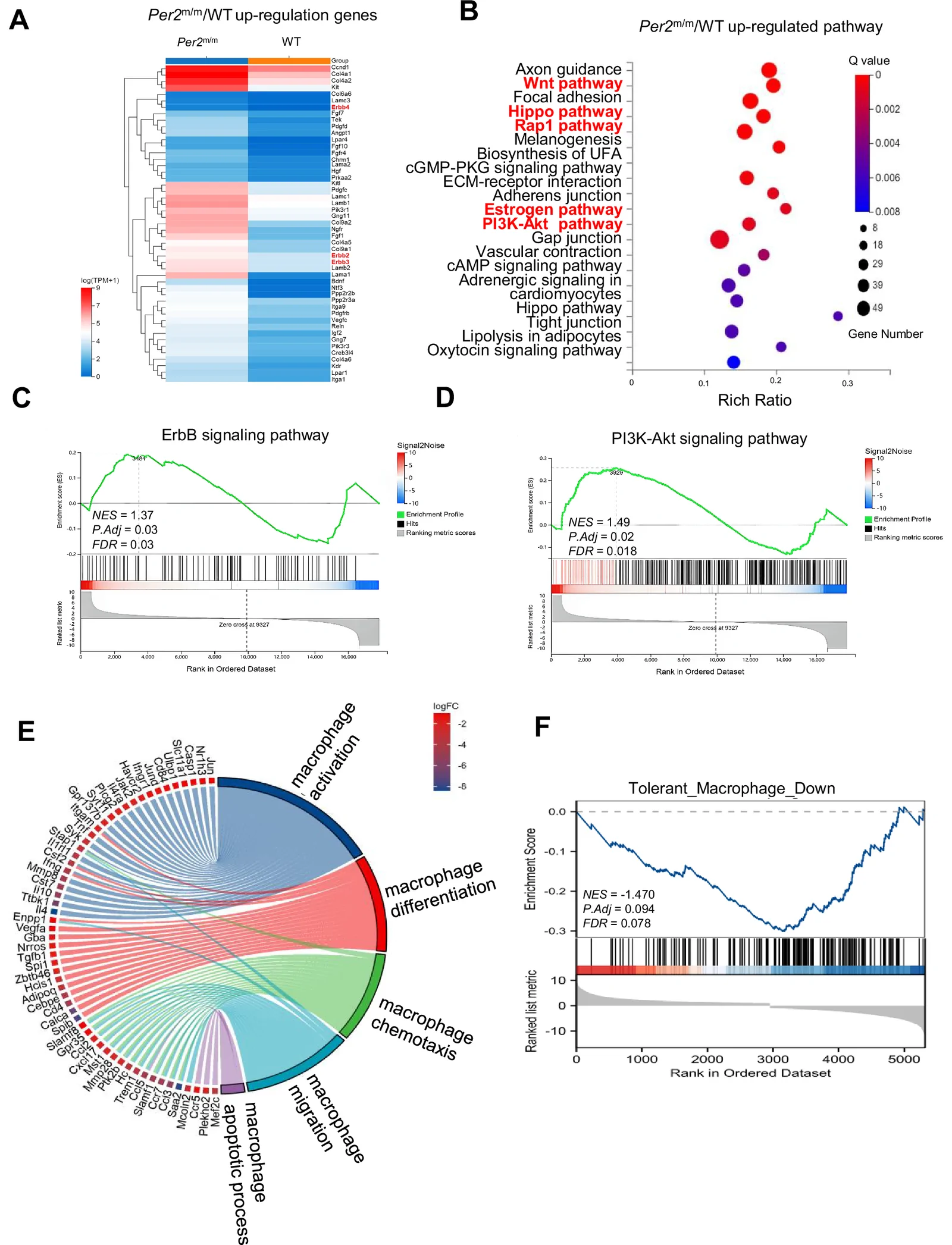
PER2 functional deficiency activates the ErbB–AKT pathway and inhibits the macrophage activation pathway. **A** Heatmap of the top 50 upregulated genes in *Per2*^m/m^ versus WT DMBA/MPA induced tumors. **B** KEGG enrichment analysis of top 21 upregulated pathways in *Per2*^m/m^ versus WT induced tumors. **C, D** ErbB2 (HER2) and PI3K-AKT pathways in *Per2*^m/m^ versus WT induced tumors (gene set enrichment analysis, GSEA). **E** Chord diagram illustrating integrated GO/KEGG and FC analysis of downregulated macrophage activation-related signaling pathways in tumors from *Per2*^m/m^ versus WT mice (The right half shows term blocks, with colors distinguishing different terms, and block size corresponding to the Counts. The chords connecting gene blocks on the left to term blocks on the right indicate which genes are associated with which terms. **F** GSEA of downregulation of tolerant macrophage activation-related gene signatures in tumors from *Per2*^m/m^ versus WT tumors.

Given that PER2 functional deficiency promotes an immunosuppressive TME (Fig. 2) and that PER2 downregulation enhances BC aggressiveness (Fig. S2), we hypothesized that PER2 regulates not only the immune microenvironment but also the intrinsic proliferative capacity. We then analyzed transcriptomic data from *Per2*^m/m^ and *Per2*^wt^ tumors, identifying 1902 upregulated and 1880 downregulated genes in *Per2*^m/m^ tumors (Fig. S4A). In addition to the upregulation of a gene set transcriptionally associated with CLOCK: MAL1 (E-box dependent gene activation, Fig. S4B), GO and KEGG enrichment analyses revealed marked activation of oncogenic signaling pathways, including Wnt, Hippo, and estrogen signaling, with strong enrichment of the ErbB-PI3K-AKT axis, a well-established driver of BC progression[55–57] (Fig. 5A, B). GSEA further confirmed the activation of ErbB signaling and PI3K-AKT pathway (Fig. 5C, D), indicating that the ErbB and PI3K-AKT pathways represent key oncogenic drivers of *Per2*^m/m^ tumor aggressiveness. In addition, genes involved in fatty acid oxidation were upregulated (Fig. S4C), suggesting a reprogrammed energy metabolism to meet the energy consumption for tumor cell proliferation.

To further elucidate the specific mechanisms by which PER2 functional deficiency promotes tumor immunosuppression, integrated GO/KEGG enrichment and fold-change analyses were performed, revealing a broad suppression of macrophage-associated immune pathways. Specifically, M1-like macrophage activation and cytotoxic programs were significantly downregulated in *Per2*^m/m^ tumors (Fig. 5E), whereas gene signatures associated with M2-like macrophage polarization were enriched (Fig. 5F). These findings are consistent with the increased infiltration of M2-like macrophages observed in the *Per2*^m/m^ TME (Fig. 2E). Moreover, T cell-related pathways, including TCR signaling and PD-1 blockade-responsive gene sets, were also downregulated (Fig. S4D, E), indicating a broad impairment of adaptive immunity. Analysis of TCGA-BRCA data further revealed a negative correlation between PER2 expression and macrophage-associated immunosuppressive signatures (Fig. S4F). Collectively, these data suggest that PER2 functional deficiency is associated with activation of the HER2-AKT pathway, together with an immunosuppressive TME characterized by M2-like macrophage polarization and impaired T cell function, suggesting a potential association between HER2-related proliferative signaling with reduced immune surveillance.

### CLOCK transactivates HER2 and CD47 to drive tumor aggressiveness and immune evasion

**Fig. 6:**
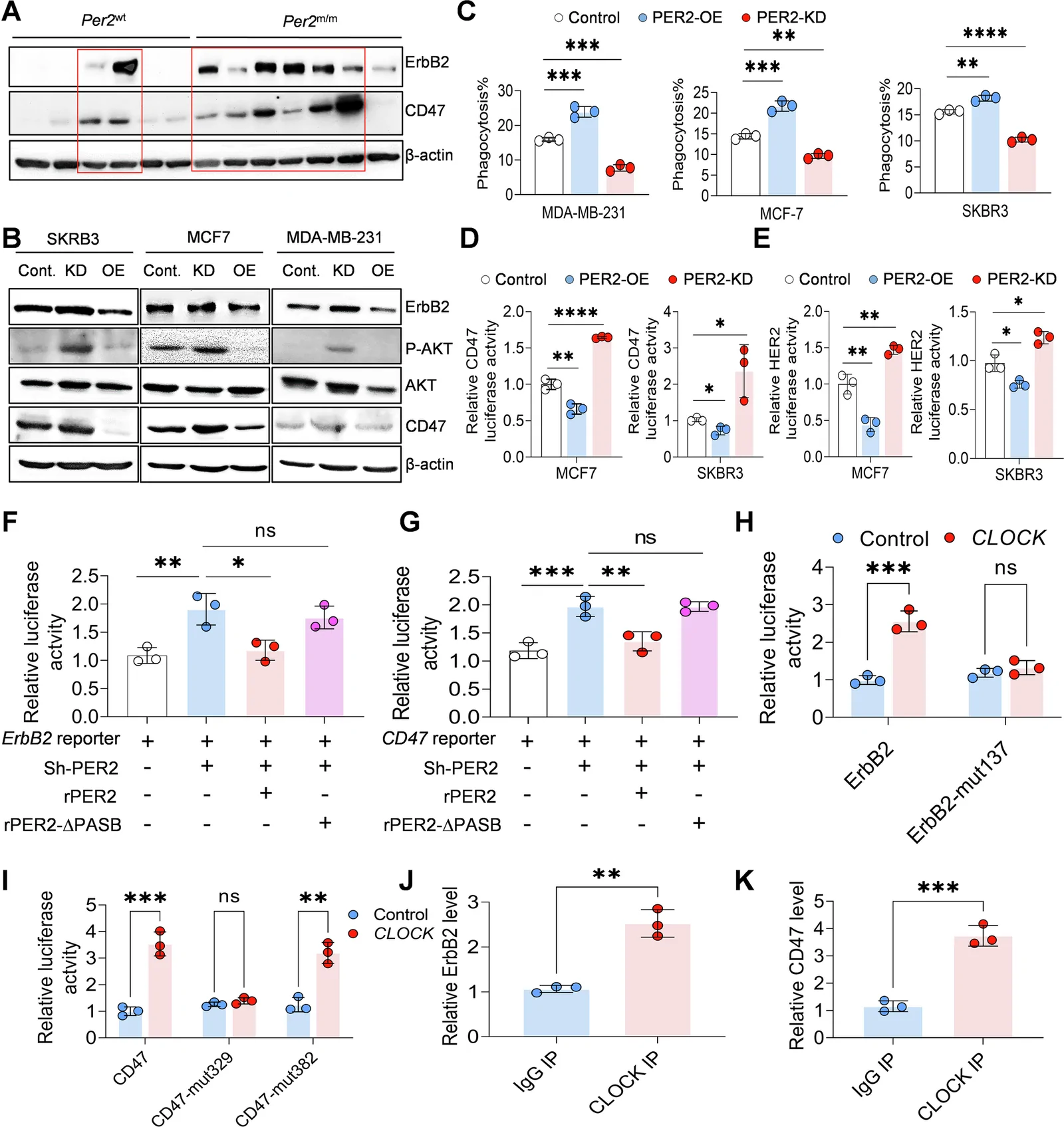
CLOCK mediates HER2/CD47-driven immune evasion upon PER2 functional deficiency. **A** Western blot of HER2 and CD47 in tumors from *Per2*^m/m^ versus WT mice in the DMBA/MPA induced model (WT mice, *n* = 6, *Per2*^m/m^ mice, *n* = 7). **B** Western blot of HER2, AKT, p-AKT, CD47, and β-actin of three BC cell lines with PER2 overexpression (PER2-OE) or inhibition (PER2-KD). **C** Macrophage phagocytosis on BC cells with PER2-OE or PER2-KD (one-way ANOVA test). **D** CD47 Luc-reporter activity in BC cells with PER2-OE or PER2-KD (one-way ANOVA test). **E** HER2 Luc-reporter activity in BC cells with PER2-OE or PER2-KD (one-way ANOVA test). **F, G** HER2 and CD47 promoter-controlled Luc-reporter activity in MCF-7 cells indicates reporter combinations (one-way ANOVA test). **H, I** Activities of HER2 and CD47 Luc-reporters with CLOCK binding motif wild type or mutation (two-tailed Student’s *t* test). Chromatin immunoprecipitation was performed using Flag antibody in MCF-7 cells transfected with Flag-CLOCK plasmid, and enrichment of CLOCK at specific binding sites in the HER2 (**J**) or CD47 (**K**) promoter was quantified by RT-qPCR (two-tailed Student’s *t* test). In **C–K**, *n* = 3, data shown as mean ± SD, ns, *p* > 0.05; *p < 0.05; **p < 0.01; ***p < 0.001; ****p < 0.0001.

Previous studies have demonstrated that HER2 signaling promotes BC aggressiveness [56], while activation of the HER2-AKT axis further enhances CD47 expression, leading to coordinated HER2/CD47 co-expression [41]. Given the concurrent activation of the HER2–AKT signaling axis identified in *Per2*^m/m^ tumors by RNA-seq analysis (Fig. 5), together with the M2 macrophage–dominant immunosuppressive TME observed in *Per2*^m/m^ tumors (Figs. 2, 3), we hypothesized that the aggressive phenotype associated with PER2 functional deficiency may be mediated by coordinated upregulation of HER2 and CD47. Indeed, co-expressing HER2 and CD47 were more frequently detected in the *Per2*^m/m^ tumors (5/6) than *Per2*^wt^ counterparts (2/6) (Fig. 6A). In BC cells, *PER2*-KD upregulated both HER2 and CD47, whereas *PER2* overexpression (PER2-OE) suppressed both genes (Figs. 6B and S5A, B). Moreover, AKT Ser473 phosphorylation was markedly increased in *PER2*-KD cells but reduced in *PER2*-OE cells (Fig. 6B), suggesting that tumor-cell–intrinsic PER2 downregulation is associated with activation of the HER2–AKT signaling axis, accompanied by coordinated HER2/CD47 upregulation.

Consistent with the established role of CD47 in inhibiting macrophage-mediated phagocytosis [58], macrophage phagocytosis was significantly reduced in *PER2*-KD BC cells but enhanced in *PER2*-OE cells, in accordance with corresponding CD47 expression levels (Figs. 6C and S5C–E). In addition, transcription of both HER2 and CD47 reporters was inhibited by *PER2*-OE and enhanced by *PER2*-KD (Fig. 6D, E). Because PER2 functions as a transcriptional repressor of CLOCK: BMAL1-mediated transcriptional activation, we next investigated whether CLOCK directly regulates HER2 and CD47 transcription. *Per2*^m/m^ mice harbor a deletion of the *PAS-B* domain required for PER2–CRY complex formation and repression of CLOCK:BMAL1 transcriptional activity [51]. Rescue experiments further demonstrated that reconstitution with PER2-WT, but not the PER2-ΔPAS-B mutant, suppressed HER2 and CD47 promoter activation (Fig. 6F, G). JASPAR analysis identified putative E-box motifs within the HER2 and CD47 promoters that could potentially bind the CLOCK:BMAL1 heterodimer (Fig. S6A–C). CLOCK overexpression markedly increased the transactivation activity, which was abolished by mutation of the predicted binding elements (HER2-137 site, CD47-329 site; Figs. 6H, I and S6D, E). ChIP-qPCR further confirmed CLOCK occupancy at these promoter regions (Fig. 6J, K). Furthermore, RNA-seq analysis of CLOCK-knockdown MCF7 cells revealed downregulation of CD47 and extracellular matrix-related signaling pathways (Fig. S7A, B), with corresponding protein-level validation showing reduced CD47 and phosphorylated AKT (Fig. S7C). Together, these findings suggest HER2 and CD47 as two key downstream effectors of PER2 functional deficiency mediated by aberrant CLOCK activation. These alterations may contribute to BC progression associated with PER2 functional deficiency through coordinated HER2-driven proliferation with CD47-mediated anti-phagocytic immune evasion.

## Discussion

This study identifies a potential protumor signaling axis involving PER2-CLOCK-HER2/CD47, which provides insight for BC incidence and progression due to dysfunction of a key clock gene. Increasing evidence suggests that CRD is associated with BC progression and promotes an immunosuppressive TME [14, 21, 59]. Given the tumor-suppressive role of PER2 in the circadian clock system, we investigated the impact of PER2 functional deficiency on tumor progression and immune suppression. Our findings suggest that PER2 functional deficiency promotes BC progression and immune suppression. Mechanistically, PER2 downregulation enhanced CLOCK-dependent transcriptional activity and increased the expression of HER2 and the immune checkpoint molecule CD47, suggesting a potential connection between circadian gene disruption, oncogenic signaling, and immune evasion. These findings suggest that impaired PER2 function promotes tumor aggressiveness through both tumor cell-intrinsic mechanisms and immune-modulatory mechanisms.

Our findings support a critical role for PER2 in maintaining systemic antitumor immunity during BC progression. Reciprocal tumor–host transplantation experiments demonstrate that *Per2*^m/m^ tumors grew more rapidly in *Per2*^m/m^ hosts than in WT hosts, indicating that host PER2 functional status strongly influences tumor progression and immune surveillance. *Per2*^m/m^ host TME exhibited reduced cytotoxic CD8⁺ T cells and M1 macrophages, with enrichment of Tregs and M2 macrophages, features characteristic of an immunosuppressive tumor milieu. Similar to the immunosuppressive remodeling induced by *Per2*^m/m^ mice, tumors in *Bmal1*^−/−^ mice also exhibit reduced CD8^+^ T cell infiltration and an overall immunosuppressive TME [21]. These phenotypes closely resemble those observed in light-induced CRD models, suggesting that systemic circadian dysregulation compromises immune surveillance [13, 60]. Clinically, these findings may help explain the increased BC incidence, metastasis, and poor prognosis associated with CRD [14, 61, 62]. Previous studies have shown that the *Per2* PAS-B mutation disrupts core clock gene expression programs and impairs circadian transcriptional regulation [28, 29]. The PER2 functional deficiency mouse model used in the present study also carries a mutation within the PAS domain of PER2, which disrupts the interaction between PER2 and the CLOCK:BMAL1 complex and impairs the circadian transcriptional repressive function of PER2 [51]. Therefore, the intrinsic tumor-promoting function coordinated with the immunosuppressive TME driving tumorigenesis and progression associated with PER2 functional deficiency. Our data reflect the consequences of PER2 functional deficiency in a genetic circadian context, rather than direct modeling of behavioral or environmental CRD. Consistent with previous studies demonstrating that CRD contributes to an immunosuppressive TME [13, 60], our results extend these observations by highlighting a role for *Per2*-dependent circadian regulation in tumor–immune interactions. However, this study does not distinguish the relative contributions of systemic circadian dysregulation and tumor-intrinsic effects. Future studies elucidating the immune compartments and comparison to environmental CRD (e.g., jet lag) will provide evidence to define the systemic role of PER2 in tumor progression and immune remodeling.

Functionally, our findings support a tumor-intrinsic suppressive role for PER2 in BC. Clinically, PER2 expression is significantly reduced in tumors from patients with poor prognosis, and low PER2 expression is associated with unfavorable clinical outcomes in BC. PER2 knockdown significantly enhanced proliferation, invasion, and colony formation, whereas PER2 overexpression suppressed these malignant phenotypes. In vivo, orthotopic transplantation of PER2-knockdown tumor cells accelerated tumor growth and promoted immunosuppressive remodeling of the tumor microenvironment. Emerging evidence indicates that PER2 is a tumor-intrinsic suppressor through multiple pathways. PER2 inhibits the transcription of EMT-related genes such as TWIST1 and SLUG, whereas hypoxia-induced PER2 downregulation promotes EMT in BC [37]. In addition, PER2 suppresses PD-L1 expression by inhibiting p65 nuclear translocation in the NF-κB pathway. Similarly, reduced PER2 expression in oral squamous cell carcinoma (OSCC) is associated with elevated PD-L1 expression and enhanced immune evasion [38]. Together, these findings, along with our findings, indicate that PER2 downregulation promotes tumor progression and immune evasion through coordinated regulation of tumor-promoting pathways and immune checkpoint signaling, supporting a tumor-intrinsic suppressive role for PER2.

Interestingly, in tumor-free mice, PER2 functional deficiency caused by PAS-B mutation does not significantly alter splenic immune cell composition, but is associated with impaired immune cell function, suggesting that PER2 functional deficiency primarily affects immune responsiveness rather than immune cell abundance (Fig. 4). Consistently, similar findings in *Per1/Per2* double-knockout mice show stable CD4⁺ and CD8⁺ T cell proportions at early ages, with reductions observed only at later stages, indicating that circadian gene dysfunction may exert time-dependent effects on immune homeostasis [63]. Under tumor-bearing conditions, increased expression of immunomodulatory cytokines such as TGF-β and IL-10 in tumors from *Per2*^m/m^ mice may contribute to a suppressive tumor microenvironment [64], although these effects likely reflect local tumor-associated signaling rather than systemic immune suppression. Importantly, previous studies have shown that PER2 is involved in immune regulation, including CD4^+^ T cell differentiation [36]. Consistent with these findings, our results demonstrate that immune cells isolated from *Per2*^m/m^ mice exhibit markedly reduced antitumor activity, suggesting that PER2 contributes to the maintenance of immune effector function. This functional impairment may weaken immune surveillance and increase susceptibility to tumor development. However, the precise molecular mechanisms by which PER2 regulates immune cell function remain to be fully elucidated and warrant further investigation.

Our transcriptomic profiling revealed activation of the HER2-AKT signaling in tumors from *Per2*^m/m^ mice. Previous studies have reported that HER2/AKT activation can increase CD47 expression and contribute to immune evasion [41]. HER2 activation enhances PI3K-AKT-mTOR signaling to promote proliferation, while CD47 engages SIRPα on macrophages to deliver a “don’t eat-me” signal, facilitating immune evasion [41], highlighting the synergistic nature of proliferative and immune-evasive pathways. In our study, enhanced CLOCK activity under PER2 functional deficiency conditions was accompanied by increased HER2 and CD47 expression. These findings support a model in which both PER2 functional deficiency and downregulation enhance CLOCK:BMAL1-mediated transcriptional activity, leading to increased HER2 and CD47 expression. In addition, HER2-AKT signaling may further contribute to CD47 upregulation through a potential feed-forward mechanism. The concurrent activation of proliferative signaling and immune-associated pathways may contribute to the aggressive phenotype observed in tumors from *Per2*^m/m^ mice. However, the present study does not directly demonstrate the functional roles of the HER2-AKT or CD47 pathways in tumor progression or in vivo remodeling of the TME under PER2 functional deficiency conditions. Therefore, the biological and therapeutic significance of concurrent HER2 and CD47 targeting in PER2-dysregulated tumors remains to be determined in future functional studies.

Prior work has shown that the CLOCK:BMAL1 complex drives tumorigenesis by upregulating non-circadian genes such as Wee1 and HIF1α [65, 66]. Also, in GBM, CLOCK: BMAL1 transcriptionally promotes OLFML3 expression and tumor immune evasion [67]. Together with our findings, these studies support an association between abnormal CLOCK:BMAL1 transcriptional activity and tumor-promoting or immune-related gene expression programs. Meanwhile, our previous analysis of the TCGA BRCA dataset showed a positive correlation between CLOCK and HER2 expression [68], suggesting a potential link between CLOCK and HER2-driven tumor biology. Consistently, epidemiological studies indicate that CRD, such as night-shift work, is associated with an increased risk of HER2-positive BC [69]. These findings together implicate a potential connection between CLOCK dysregulation and HER2 signaling. Given that both HER2 and CD47 promoters contain canonical E-box elements, aberrant CLOCK:BMAL1 activity may play a key role in regulating additional oncogenic and immune-related genes in cancer.

In summary, PER2 functional deficiency was associated with increased HER2/AKT signaling, elevated CD47 expression, and an immunosuppressive TME. These findings support a potential link between circadian disruption and aggressive BC phenotypes and suggest that the PER2-CLOCK-HER2/CD47 axis may serve as a potential therapeutic target. Future studies should aim to validate PER2-associated metabolic reprogramming in larger clinical cohorts, characterize PER2 dynamics in systemic immunity and tumor-infiltrating lymphocytes, and define temporal windows of therapeutic vulnerability for circadian-guided interventions against BC.

## Supplementary information

Supplementary Data

## Data Availability

All data, including the *Per2*^m/m^ tumor transcriptome data referenced during the study, are available from the corresponding author upon reasonable request. The data supporting this study’s findings are within the Article, Supplementary Information, or information available from the corresponding author upon reasonable request. Source data are provided in this paper. Requests for resources and reagents should be directed to Jian Jian Li (jijli@ucdavis.edu).
