# Supplementary material for: Functional deficiency of circadian regulator PER2 enhances breast cancer susceptibility and progression with immunosuppression
Source: Oncogene. 2026 Aug 12;45(40):4269–83. doi: 10.1038/s41388-026-03941-3 (PMC13600920; doi:10.1038/s41388-026-03941-3)
Supplement: Supplementary file 1 [file 41388_2026_3941_MOESM1_ESM.pdf]

Supplementary Data

Functional deficiency of circadian regulator PER2 enhances breast cancer susceptibility and progression with immunosuppression

Yixin Duan, Aris T. Alexandrou, Jie Huang, … , Loning Fu, and Jian Jian Li

Supplemental Figure: Figure 1-7

Supplemental Table: Table S1

Fig. S1

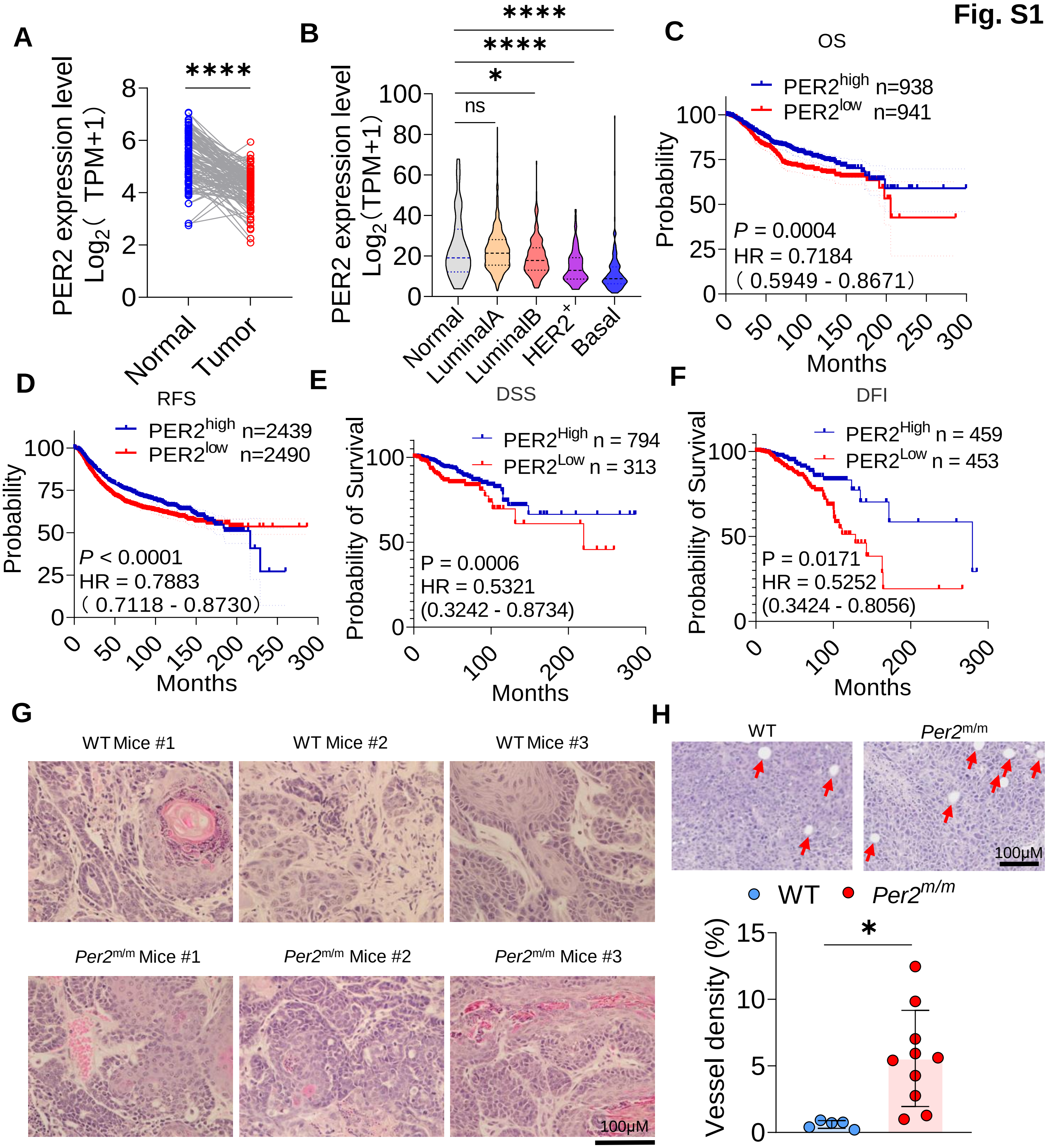

Supplemental Fig.1 PER2 is associated with breast cancer poor prognosis. A *PER2* expression in BC compared to surrounding normal tissues from TCGA BRCA cohort (n = 113, Pairwise Sample t-test). **B** PER2 expression in normal breast epithelium and different BC subtypes based on PAM50 in the TCGA BRCA cohort, Kruskal-Wallis, followed by Dunn's multiple comparisons test. **C, D** Overall Survival (OS) and Relapse-Free Survival (RFS) correlated with BC *PER2* levels using the Kaplan-Meier Plotter database. **E, F** Disease-specific survival (DSS) and disease-free interval (DFI) in breast cancer patients with high or low expression of PER2 in the TCGA-BRCA database**. G** Represent HE image and diagnosis of spontaneous breast tumors in WT and *Per2*$^{m/m}$ mice (400X). **H** Vascular area was quantified as the percentage of the total tissue area occupied by blood vessels in three random fields per sample,red arrows indicate blood vessels. Data are shown as mean ± SD. *$p < 0.05$. Scale bar = 100 μm.

Fig. S2

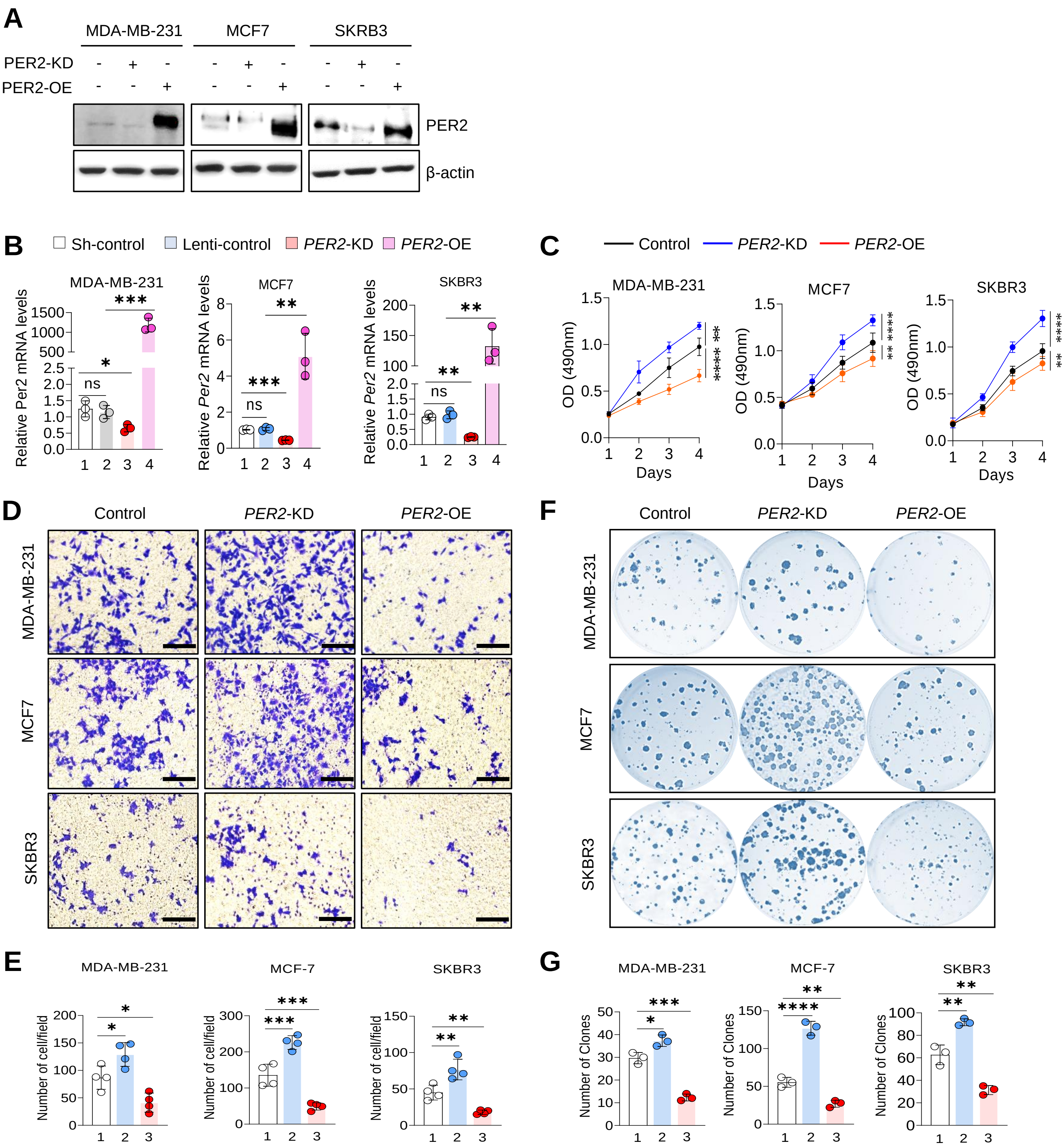

Supplemental Fig. 2. BC cell aggressiveness is related to PER2 status. PER2 protein (**A**) and mRNA (**B**) of PER2 overexpression (PER2-OE) or PER2 knockdown (Sh-PER2, PER2-KD) in three BC cell lines. Cell proliferation (**C**) and Matrigel matrix invasion tests (**D**) of PER2-OE and PER2-KD cells (scale bar= 100 µm) with quantitation (**E**). Images of clonogenicity (**F**) and quantitation (**G**) of clone formation ability. In B-H, *$p < 0.05$; **$p < 0.01$; ***$p < 0.001$, ****$p < 0.0001$.

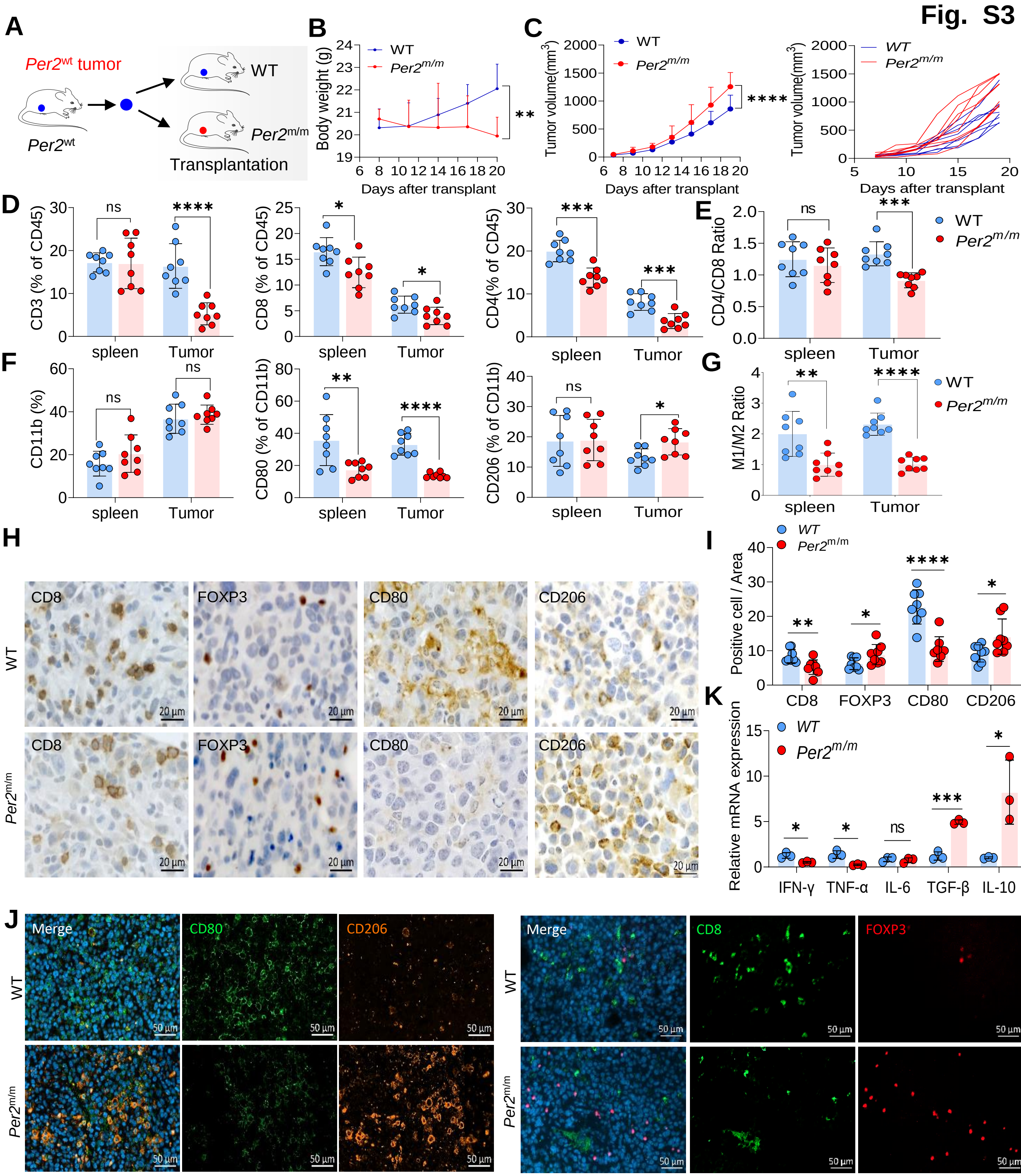
Fig. S3
A
Per2wt tumor
Per2wt
WT
Per2m/m
Transplantation
B
Body weight (g)
WT
Per2m/m
Days after transplant
C
Tumor volume(mm3)
Days after transplant
D
CD3 (% of CD45)
CD8 (% of CD45)
CD4(% of CD45)
spleen
Tumor
E
CD4/CD8 Ratio
F
CD11b (%)
CD80 (% of CD11b)
CD206 (% of CD11b)
G
M1/M2 Ratio
H
CD8
FOXP3
CD80
CD206
20 µm
I
Positive cell / Area
K
Relative mRNA expression
IFN-γ
TNF-α
IL-6
TGF-β
IL-10
J
Merge
50 µm

Supplemental Fig. 3. Tumor immune microenvironment in WT and *Per2*$^{m/m}$ mice carrying *Per2*^{wt} tumors. **A** Scheme of syngeneic mouse E0771 tumors formed in WT mice transplanted into WT and *Per2*$^{m/m}$ mice, and body weight (**B**) and tumor growth (**C**) were measured (two-way ANOVA test). Flow cytometry analysis of tumor $CD3^+$ (left), $CD8^+$ (middle), and $CD4^+$ T cells (right) in the spleens and tumors (**D**) and the $CD8^+/CD4^+$ cell ratio (**E**) in the spleens and tumors of WT and *Per2*$^{m/m}$ mice bearing E0771 tumors. **F** Flow cytometry analysis of tumor $CD11b^+$ monocytes (left), CD80+ M1 (middle), and CD206+ M2 macrophages (right) in the spleens and tumors of WT and *Per2*$^{m/m}$ mice bearing E0771 tumors. **G** $CD80^+$ M1/CD206+ M2 macrophages ratio in spleen and tumors of WT and *Per2*$^{m/m}$ mice bearing E0771 tumors (two-tailed Student's *t*-test). **H** Representative IHC images of $CD8^+$, $FOXP3^+$, $CD80^+$, and $CD206^+$ cells infiltrated in E0771 tumors in WT and *Per2*$^{m/m}$ mice**. I** Quantitation of $CD8^+$, $FOXP3^+$ $CD80^+$, and $CD206^+$ cells infiltrated in E0771 tumors in WT and *Per2*$^{m/m}$ mice**. J** Immunofluorescence of infiltrated CD8 and FOXP3 or CD80 and CD206 in E0771 tumors of WT and *Per2*$^{m/m}$ mice. **K** mRNA expression of intra-tumor cytokines IFN-γ, TNF-α, TGF-β, IL-6 and IL-10 in E0771 tumors of WT and *Per2*$^{m/m}$ mice (WT mice n = 8, *Per2*$^{m/m}$ mice n = 8, ns p >0. 05, *p < 0. 05, **p < 0. 01, ***p < 0. 001, ****p < 0. 0001).

Fig. S4

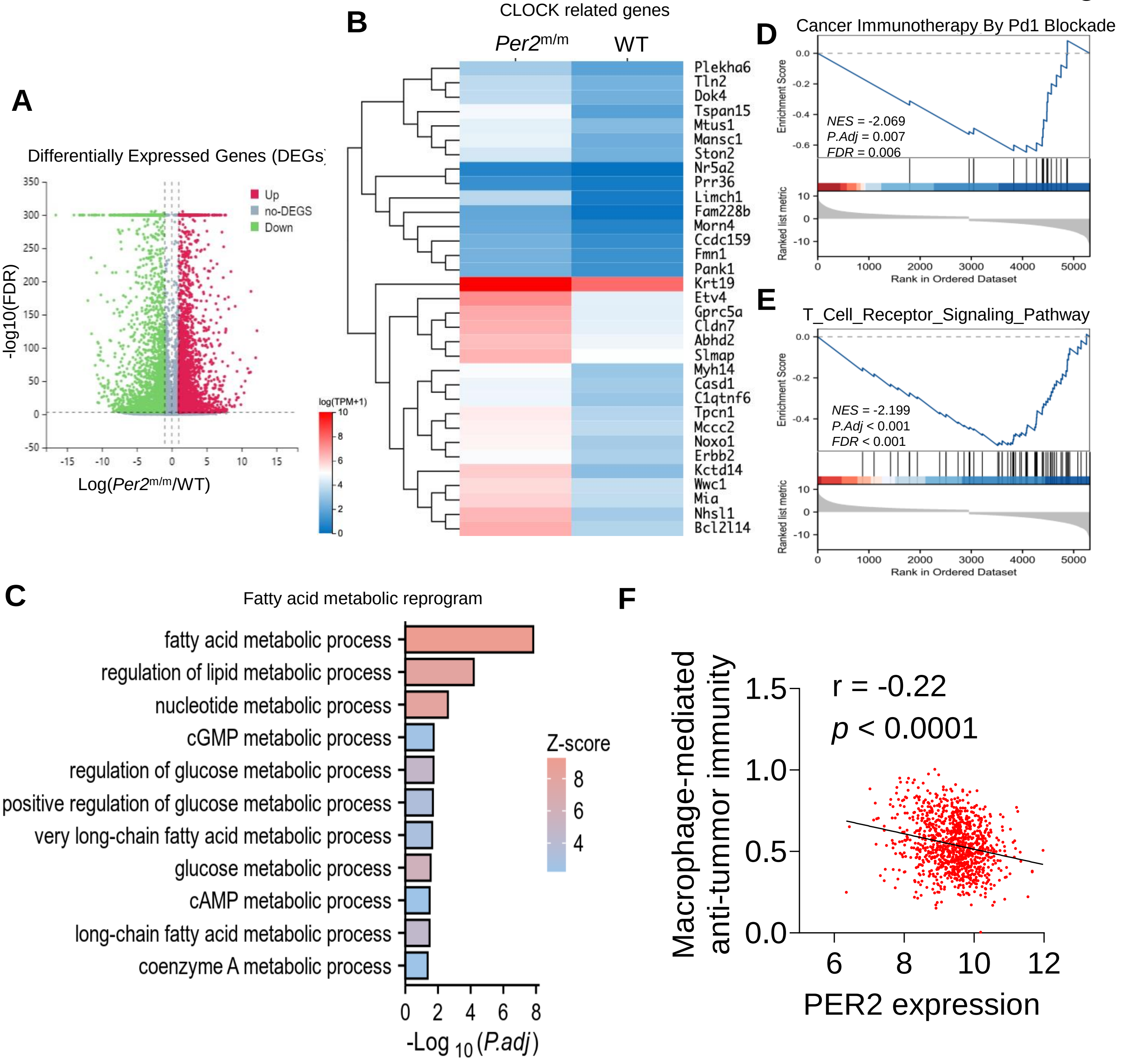

Supplemental Fig. 4. The interplay between PER2 and tumor metabolism/immunity.

A Volcano plot of transcriptomes of DMBA/MPA induced *Per2*$^{m/m}$ versus WT tumors. **B** Heatmap presentation of a cluster of CLOCK-targeted genes in *Per2*$^{m/m}$ versus WT tumors. **C** Integrated GOKEGG and FC analysis of upregulated metabolism-related signaling pathways in *Per2*$^{m/m}$ versus WT tumors. Gene set enrichment analysis(GSEA) of cancer immunotherapy by Pd1 blockade gene set (**D**)and T cell receptor signaling pathway genes(**E**) in *Per2*$^{m/m}$ versus WT tumors.

Fig. S5

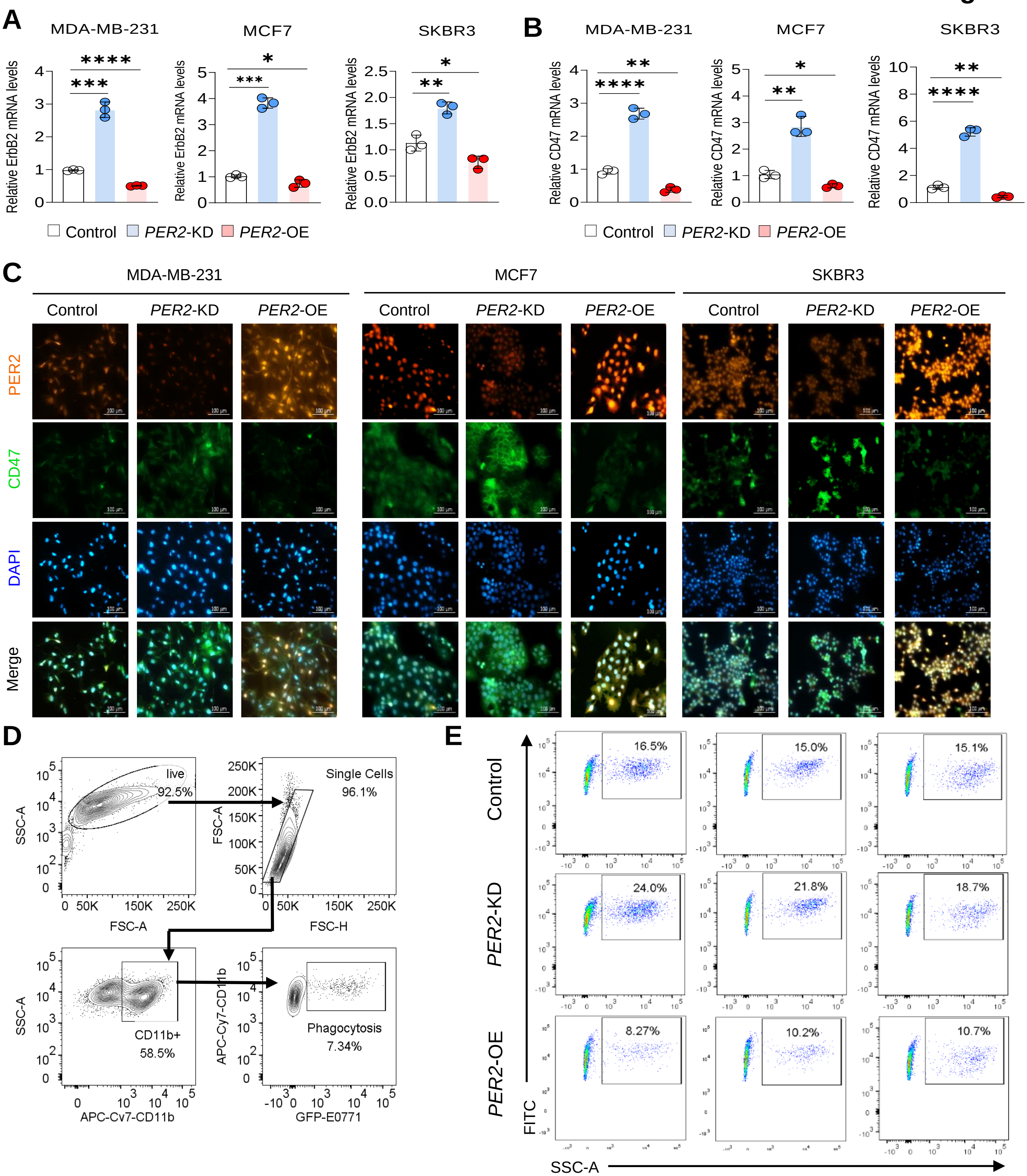

Supplemental Fig. 5. Co-expression of ErBb2 and CD47 is related to PER2 status. ErBb2 (**A**) and CD47 (**B**) mRNA measured in PER2-OE or PER2-KD cells. (n = 3, β-actin was used as an internal control). **C** Immunofluorescence (IF) of CD47 and PER2 in PER2-OE or PER2-KD (sh-PER2) cells (bar = 100 μm). **D** Gating strategy of macrophage phagocytosis, CD11b+/ GFP+ positive cells assess as phagocytosed cells. **E** FACS images for measuring macrophage phagocytosis on the PER2-OE or PER2-KD cells. In **A**, **B** and **D**, n = 3, *$p < 0.05$; **$p < 0.01$; ***$p < 0.001$; **** $p < 0.0001$.

A

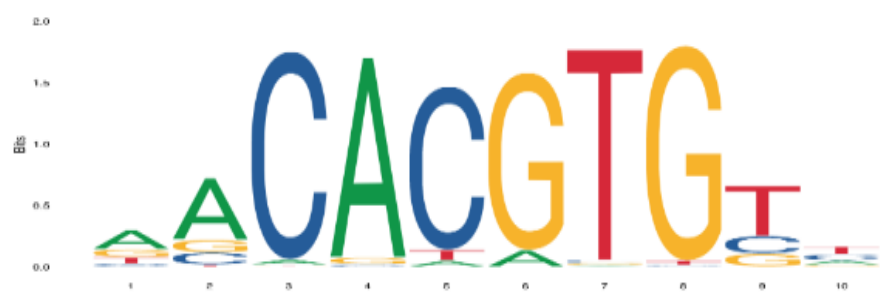

B

***CD47* promoter-CLOCK binding site**

Matrix ID	Name	Score	Relative score	Predicted sequence
MA0819.1	MA0819.1.CLOCK	5.9365144	0.81236671	GGCGCGTGCC
MA0819.1	MA0819.1.CLOCK	6.010157	0.813787109	TCCGCGTGCT

C

***ErbB2* promoter-CLOCK binding site**

Matrix ID	Name	Score	Relative score	Predicted sequence
MA0819.1	MA0819.1.CLOCK	7.707796	0.8465307	GACACCTGTC

D

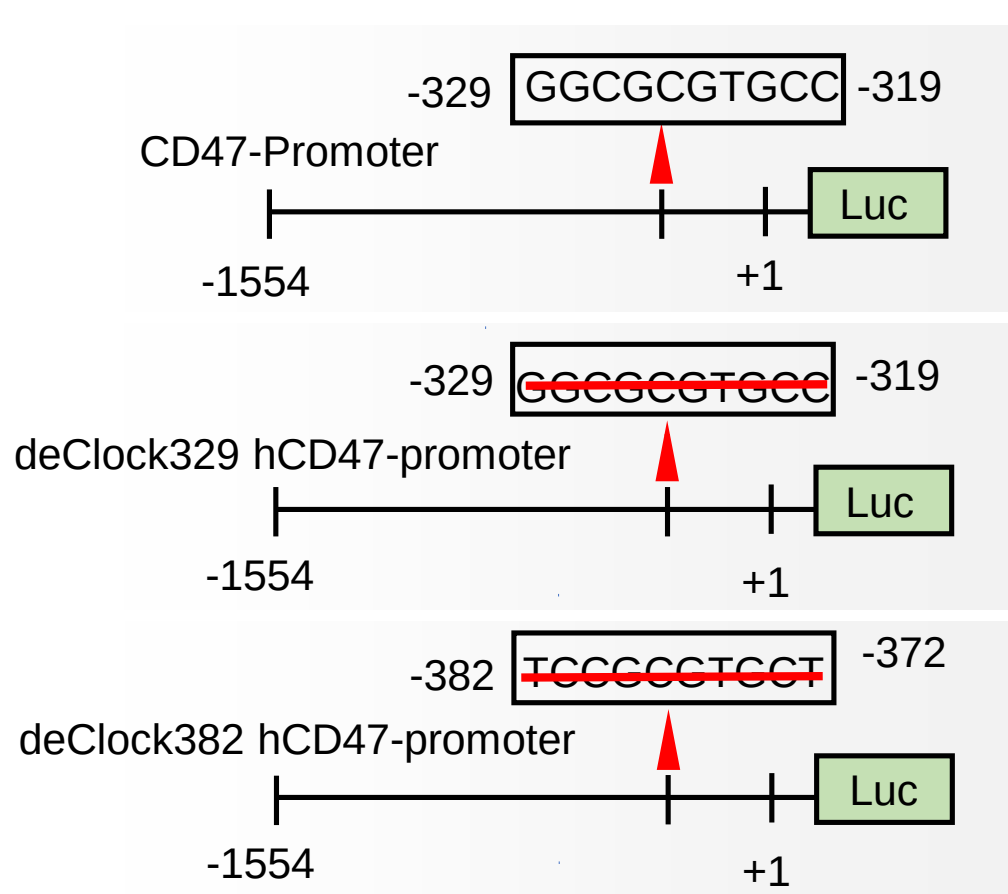

e

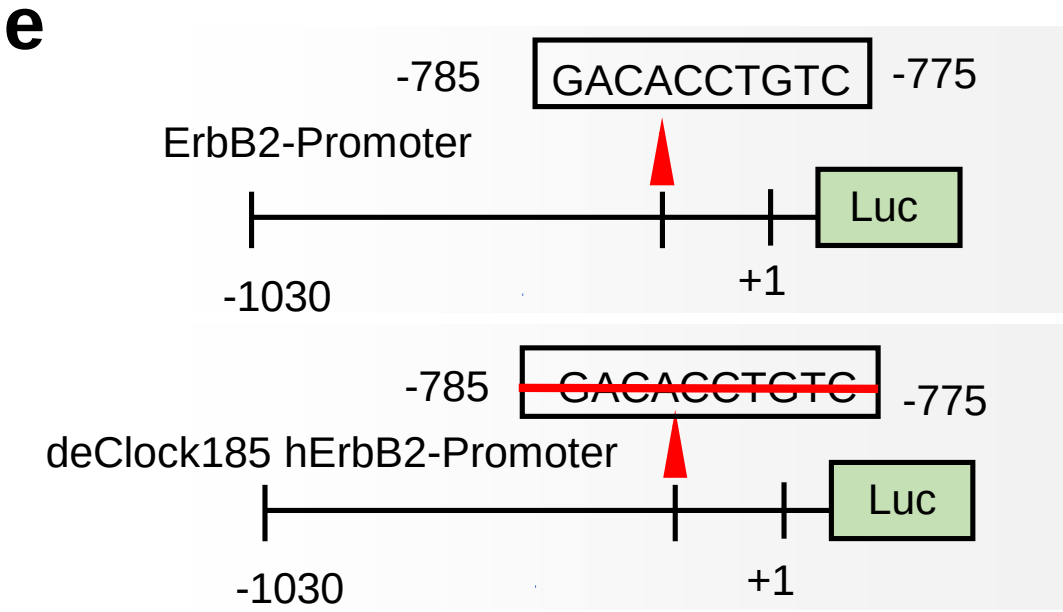

Supplemental Fig. 6. PER2-CLOCK axis is involved in ErbB2 and CD47 transcriptional regulation. A Predict CLOCK binding sequences in the transcription factor binding profile database JASPAR. **B** Predict CLOCK binding site in CD47 promotor. **C** Predict CLOCK binding site in ErbB2 promotor. **D** Scheme of creating mutations of the predicted CLOCK motif in human CD47 promoter region. **E** Scheme of creating mutations of the predicted CLOCK motif in human ErbB2 promoter region.

Fig. S7

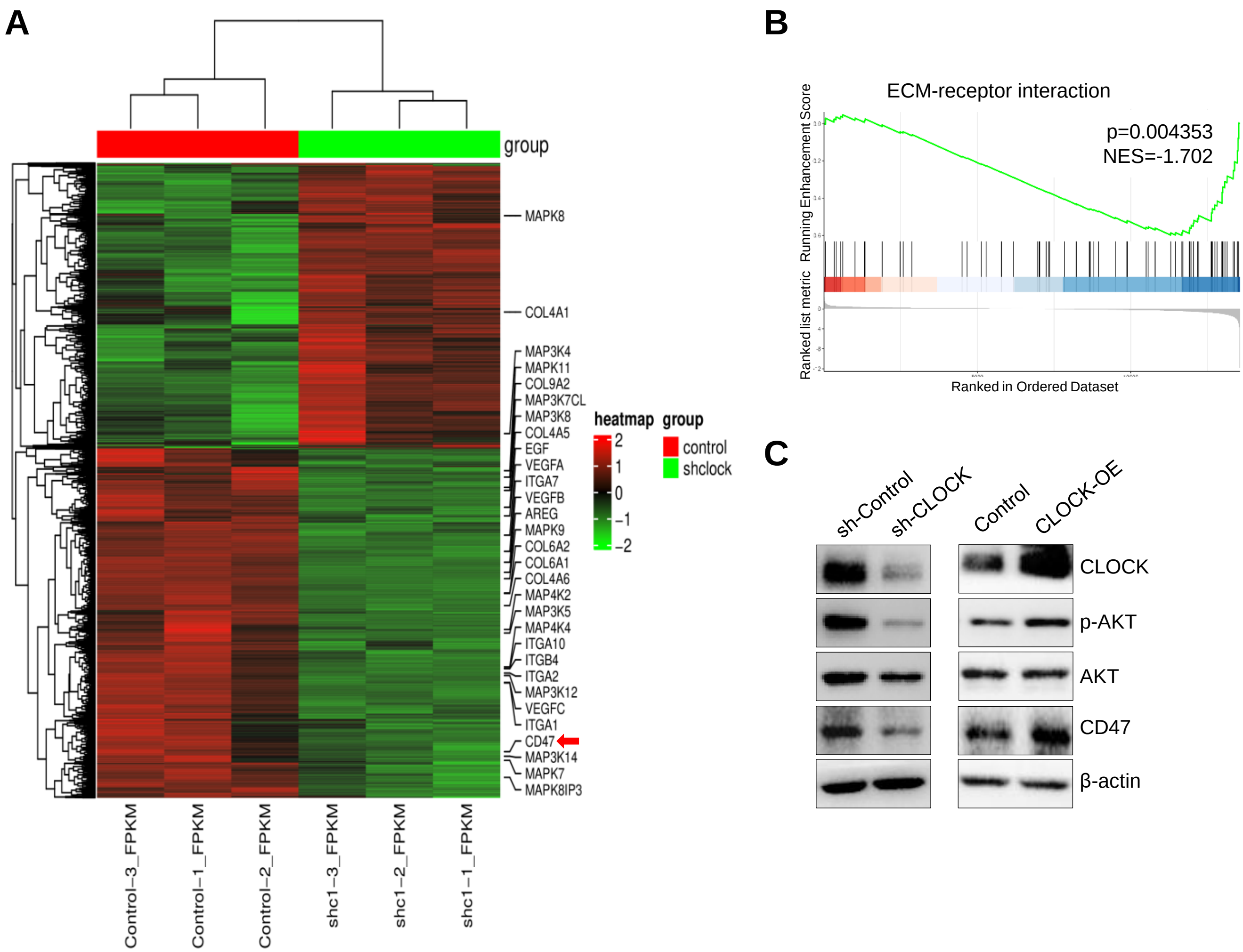

Supplemental Fig. 7. CLOCK knockdown downregulate CD47 expression. A Heatmap depict the expression of MAPK and ECM signaling genes in CLOCK-knockdown (shc1) MCF7 cells compared to the sh-Control group. **B** Gene set enrichment analysis (GSEA) was performed to compare sh-CLOCK MCF7 cells with the sh-control group, the display highlights pathways associated with ECM-signaling pathway. **C** Western blot was performed to assess the expression levels of CLOCK, p-AKT, AKT, CD47 in CLOCK-OE and sh-CLOCK MCF7 cells and their parental cells (β-actin served as the internal loading control for normalization).

Table S1 Pathologic diagnosis of induced breast tumors in WT and *Per2*$^{m/m}$ mice

Subtype	Squamous cell carcinoma	Adenocarcinoma	Adeosquamous cell carcinoma	Angiosarcoma	Pilomatrixoma
Per2$^{m/m}$	7 (46.67%）	4 (26.67%）	2 (13.3%）	1 (6.67%）	1 (6.67%）
WT	4 (60%）		2 (40%）		
